# Expression patterns of *NbrgsCaM* family genes in *Nicotiana benthamiana* and their potential roles in development and stress responses

**DOI:** 10.1038/s41598-020-66670-x

**Published:** 2020-06-15

**Authors:** Dandan Liu, Qiuying Yang

**Affiliations:** 1 State Key Laboratory for Plant Disease and Insect Pest, Institute of Plant protection, China Academy of Agricultural Sciences, Beijing, 100193 China; 2 State Key Laboratory of Biocatalysis and Enzyme Engineering, College of Life Sciences, Hubei University, Wuhan, 430062 China

**Keywords:** Genetics, Molecular biology, Plant sciences

## Abstract

*rgsCaM* has been reported as a *calmodulin-like* (*CML*) factor induced by viral infection in *Nicotiana*. There are three *CMLs* that belong to the *rgsCaM* family in *Arabidopsis thaliana*. In this study, we found a total of 5 *NbrgsCaM* coding sequences in *N. benthamiana* genome. We analyzed transcription patterns of *NbrgsCaMs* in transgenic plants expressing a β-glucuronidase (GUS) under the promoter of *NbrgsCaMs* by histochemistry staining and RT-qPCR. Similar to their *Arabidopsis* homologs, most *NbrgsCaMs* have an overlapping but distinct expression pattern in response to developmental and environmental changes. Specifically, the *NbrgsCaM4* promoter exhibited robust activity and showed distinct regulatory response to viral infection, developmental stages and other abiotic stimuli. Overall, these findings provide clues for further understanding of the *NbrgsCaM* family genes in regulating plant growth and development under biotic stress and environmental stimulation.

## Introduction

Calmodulins (CaMs) are a group of Ca^2+^ binding regulatory proteins in the signal transduction cascades of eukaryotic cells. CaMs respond to diverse biotic and abiotic stimuli, and modulate the cellular activities according to developmental and environmental changes^1^.

Plants have an extended family of CaMs, and the less conserved forms of CaMs are called CaM-related proteins or CaM-likes (CMLs)^2^. *Arabidopsis* has a large CML family including about 50 *CML* genes^3,4^. Among them, *AtCML37* (AT5G42380), *AtCML38* (AT1G76650) and *AtCML39* (AT1G76640) fall into a unique subfamily, and function in plant development, respond to stress stimuli such as hypoxia, drought and herbivore feeding^5–8^. This unique subfamily of AtCMLs are closely related to the regulator of gene silencing CML protein (rgsCaM) in *Nicotiana tabacum* (NtrgsCaM). In the first rgsCaM report, NtrgsCaM was regarded as a suppressor of virus-induced gene silencing (VIGS), which counters gene silencing effect from plants to promote viral amplification^9^. Another more recent report stated that a homologous CML in *N. benthamiana*, NbrgsCaM, also was required for suppressing VIGS through interaction with a viral suppressor of RNA silencing factor (VSR), βC1^10^. However, in publications by Tadamura^7^ and Nakahara^11^, NtrgsCaM was concluded to take an antiviral role by sequestrating the RNA silencing suppressors (RSSs) through binding of the dsRNA-binding domains of viral RSSs, acting as an antiviral pattern recognition receptor (PRR).

Morphology changes of *Nicotiana* transgenic lines over-expressing *rgsCaMs* were described to be similar to the deformities caused by viral infections in several reports, such as formation of tumors at the root-stem junction, curly leaves with wrinkles, necrosis and dwarfing^9,10,12^. However, no such changes were observed in *N. benthamiana* over-expressing *NtrgsCaM*^13^. Although these findings are rather complicated, all evidence pointed to the importance of rgsCaMs in viral infection and plant development. Furthermore, the induction of *rgsCaMs* expression by viral infection was noticed in both tobacco and *Arabidopsis*^7,10,13^, which drives us to explore their intrinsic regulation during viral infection, environmental stress and plant development, and also, the possible existence of their close homologs. Thereby, we chose *N. benthamiana*, the most widely used model plant in the virology research field^14^, to begin our study of the *rgsCaMs* regulation.

## Results

### Analysis of the sequences of *rgsCaM* family

**Figure 1:**
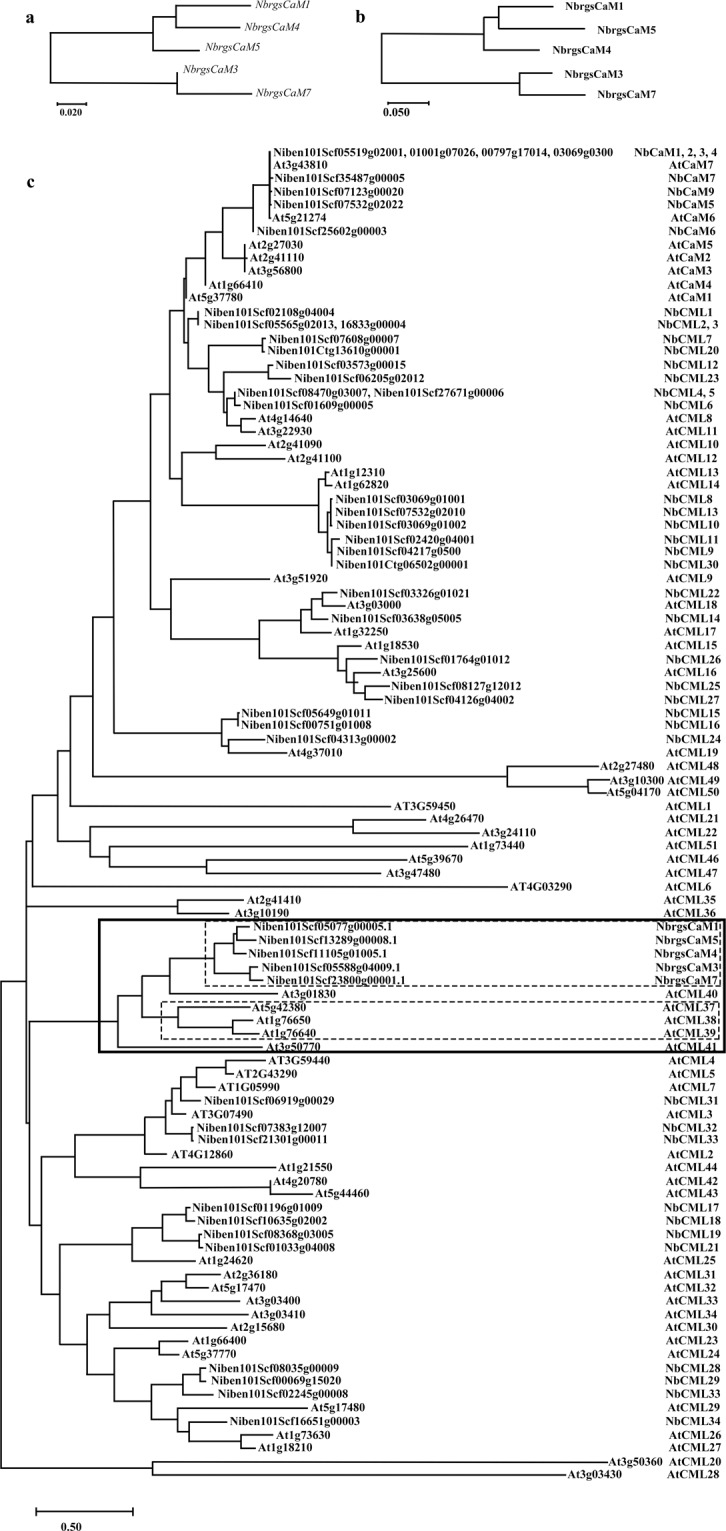
*NbrgsCaM*s form a close CML family. (**a**) The phylogenetic tree of nucleotide sequences of *NbrgsCaM*s. (**b**) The phylogenetic tree of amino acid sequences of NbrgsCaMs. c. rgsCaMs are grouped within a sub group in the CaMs and CMLs of *A. thaliana* and *N. benthamiana*. AtrgsCaM, NbrgsCaM, rgsCaM subgroups are highlighted by rectangular boxes. The scale bars represent substitutions per sequence position.

Although several *CML*s that belong to the *rgsCaM* family have been found in *Arabidopsis*, there was only one *NtrgsCaM* reported in *N. tabacum* and one *NbrgsCaM* in *N. benthamiana*^8,10,12^. As an allotetraploid^15–17^, *N. benthamiana* usually has more homologs than does *Arabidopsis*. Based on this hypothesis, we analyzed the genome of *N. benthamiana* (https://solgenomics.net/organism/Nicotiana_benthamiana/genome)^18^ in order to find all of the *rgsCaM* family genes. We found a total of 7 homologs through blasting against the *N. benthamiana* draft genome with the already known *NbrgsCaM*. These homologs were named *NbrgsCaM 1-7* according to the increasing order of scaffold serial numbers where these homologs reside (Supplementary Data S1). Due to the primitiveness of the current draft genome, some of these homologs have un-sequenced gaps. To fill these gaps, we carried out polymerase chain reactions (PCRs) with homolog-specific primers that were designed according to the available genome resources^18^, and confirmed/corrected the full-length sequences of these homologs (Supplementary Data S2). Amino acid sequences predicted from these homologs are listed in Supplementary Data S3. The newly discovered *NbrgsCaM1*, *3*, *5*, *7* have no predictable intron within the coding region, which is similar to the already published *AtCML37*, *38*, *39*, *Nt*/*NbrgsCaM* (*NbrgsCaM4* in this study is the previously reported *NbrgsCaM*). However, *NbrgsCaM2* and *NbrgsCaM6* contain only 2 fragments of the consensus *NbrgsCaMs* open reading frames (ORFs). The two fragments of *NbrgsCaM2* and *NbrgsCaM6* have ORFs less than 100aa in length (Supplementary Fig. S1 and Supplementary Data S3). There is no predicted intron-exon junction between the two fragments to bridge them into longer coding sequences (predicted on NetGene2 Server, http://www.cbs.dtu.dk/services/NetGene2/)^19^. Thus, they looked like pseudogenes that derived from full-length ancestral *rgsCaM* genes. Through blasting these ORF fragments to the online NCBI protein library^20^, we found that the shorter ORFs encode incomplete amino acid fragments of rgsCaM family proteins that do not contain any known motifs; while the longer ones encode only one EF-hand superfamily motif (Supplementary Fig. S1). As the CaM family proteins typically require a conserved pair of EF-hand superfamily motifs for their function (Supplementary Fig. S1)^21^, we proposed that *rgsCaM2* and *rgsCaM6* are either pseudogenes that do not encode functional proteins, or encode new proteins of a yet unknown function. Thus we focused on *rgsCaM1*, *3*, *4*, *5* and *7* only, to elucidate the character of *rgsCaM* family genes in *N. benthamiana*. The rooted phylograms of the coding nucleotide sequences and protein sequences show close similarity within the *NbrgsCaM* family (Fig. 1a,b). To clarify the phylogenetic relationship of these NbrgsCaMs with other CMLs, we constructed a phylogenetic tree using protein sequences of CaMs and CMLs in *N. benthamiana* and *A. thaliana* genome. NbrgsCaMs group with AtCML37, 38 and 39, which are probably AtrgsCaMs, as previously reported (Fig. 1c)^4,10,12^. However, rgsCaMs do not form a distinct branch that is separated from other CMLs. They form a sub-branch within the CML family in the phylogenetic tree (Fig. 1c). We also noticed that besides the previously reported AtrgsCaMs (AtCML37, 38 and 39), AtCML40 and 41 fall into this subgroup, indicating their close evolutionary relationship.

### Analysis of the promoters of *rgsCaM* family

Our analysis (by Softberry TSSP program online prediction, http://linux1.softberry.com/ ^22^) of the regions between the stop codon of the previous gene and the start codon of *AtCML37*, *38* and *39*, which are putative promoters named as *AtrgsCaMp*s or *AtCMLp*s, revealed promoter and enhancer motifs (listed in Supplementary Data S4) that relate to development, phytohormonal regulation and environmental stresses. The correlation between transcription factors and the predicted transcription factor binding sites/RegSites can be found in the Softberry RegSite database (http://www.softberry.com/berry.phtml?topic=regsitelist)^23^. For clarity, we did not list the candidate transcription factors for each promoter/enhancer motifs. Instead, we summarized the transcription factors for these predicted motifs (Supplementary Table S1), consistent with the reported involvement of *AtrgsCaM* in development and stress responses^5,6,8,24,25^.

The promoters of *NbrgsCaM*s also contain multiple regulatory elements (Supplementary Data S5), many of which are the same type of motifs that exist across the *rgsCaM* family. The transcription factors that are predicted for the recognition of these regulatory elements are listed, together with their specific functions (Supplementary Table S2). According to the description of these transcription factors, *NbrgsCaMp*s should be related to multiple developmental, environmental and plant hormonal regulations, and with overlapping but distinct stimuli response patterns among the *NbrgsCaMp*s (Supplementary Table S3). Thus, combining the promoter analysis of *AtrgsCaMp*s (Supplementary Table S1) and the reported function of *AtrgsCaM*s^5,6,8,24,25^, it can be deduced that *rgsCaM*s are not only homologous in coding sequences, but also respond to similar types of environmental and developmental stimuli in *Nicotiana* and *Arabidopsis*. However, the promoters of *rgsCaM*s are rather conserved in regulatory motifs but not in sequence, and the enhancer motifs do not hold the same positions (Supplementary Data S4 and S5), suggesting that the functionally conserved *rgsCaMs* evolved subtle and distinct regulatory niches to carry out precise regulatory work.

### β-glucuronidase (GUS) reporter analysis of the promoters of *rgsCaM* family genes

To analyze the expression profile of *rgsCaMs*, we constructed chimeric *rgsCaM-promoter::GUS* (*rgsCaMp::GUS*) reporters. GUS staining of the transgenic *N. benthamiana* with *rgsCaMp::GUS* reporters was carried out at various developmental stages and under specific stress treatments.

**Figure 2:**
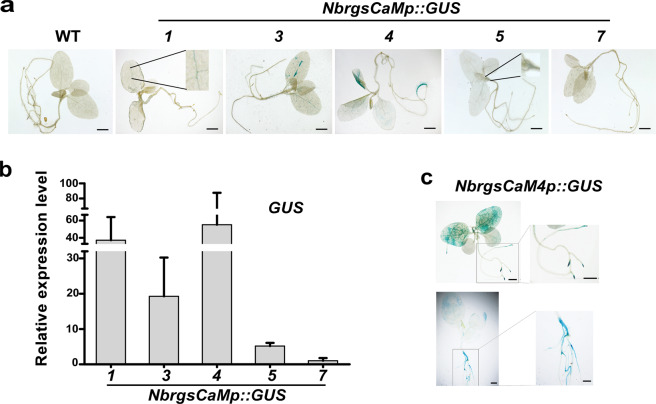
*NbrgsCaMp*s have various levels of activity. (**a**) GUS histochemistry analysis revealed various promoter activities of *NbrgsCaM*s in seedlings of the *NbrgsCaMp::GUS* transgenic *N. benthamiana*. (**b**) RT-qPCR results of the GUS expression level confirmed the histochemistry results. The relative GUS expression level of *NbrgsCaMp7::GUS* transgenic *N. benthamiana* plants was set as 1, as it showed no detectable GUS staining. The relative GUS expression levels of the other *NbrgsCaMp::GUS* transgenic *N. benthamiana* plants were calculated using that of the *NbrgsCaMp7::GUS* transgenic *N. benthamiana* plants as control. (**c**) The promoter activity of *NbrgsCaM4* is tissue-specific. The GUS staining accumulates mostly in veins and roots. Bars = 5 mm.

**Figure 3:**
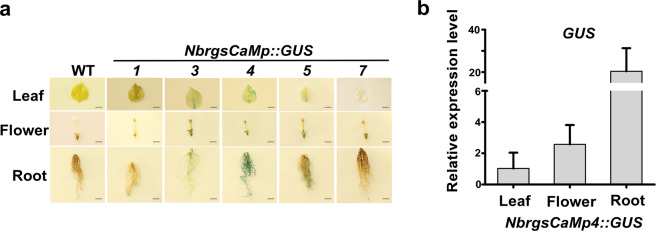
*NbrgsCaMp*s display tissue-specific activities. (**a**) Gus histochemistry analysis revealed tissue-specific activities of *NbrgsCaMp*s in the *NbrgsCaMp::GUS* transgenic *N. benthamiana*. Bars= 5 mm. (**b**) RT-qPCR results of the GUS expression levels confirmed the histochemistry results.

The transgene of *rgsCaMp::GUS* reporters (*NbrgsCaMp1::GUS*, *NbrgsCaMp3::GUS*, *NbrgsCaMp4::GUS*, *NbrgsCaMp5::GUS* and *NbrgsCaMp7::GUS*) afflicted no impact on the growth of *N. benthamiana*, indicating that our reporter system didn’t intervene with the physiology of transgenic plants. The GUS staining in these transgenic plants can actually reflect the endogenous promoter activity of these *NbrgsCaMp*s. In seedlings of the *N. benthamiana* transgenic lines, GUS expression was detected in *NbrgsCaMp1::GUS*, *NbrgsCaMp3::GUS*, *NbrgsCaMp4::GUS* and *NbrgsCaMp5::GUS*, while *NbrgsCaMp7::GUS* showed no observable staining at all (Fig. 2a). Among the transgenic plants with GUS expression, *NbrgsCaMp3::GUS* and *NbrgsCaMp4::GUS* showed darker GUS staining than *NbrgsCaMp1::GUS* and *NbrgsCaMp5::GUS* (Fig. 2a). The GUS transcription level was further measured by reverse transcription quantitative polymerase chain reaction (RT-qPCR). The RT-qPCR result affirmed that the GUS staining indeed reflected the transcription level that is the indicator of promoter activity (Fig. 2b). As the seedlings grew older, we found that more GUS staining accumulated in the root of *NbrgsCaMp4::GUS* transgenic *N. benthamiana* plants (Fig. 2c), revealing that *NbrgsCaMp4* activity is developmental stage- and tissue-associated. To systemically analyze the *NbrgsCaMps* activity, we carried out GUS staining assays in various plant organs. The results showed that, in mature leaves, *NbrgsCaMp3* and *NbrgsCaMp4* were the most robust promoters among all of the *NbrgsCaMps*, while *NbrgsCaMp1* and *NbrgsCaMp5* were much weaker, whereas the GUS staining in *NbrgsCaMp7::GUS* was almost undetectable. Likewise, in flowers, the promoter activity was robust for *NbrgsCaMp3*, *NbrgsCaMp4* and *NbrgsCaMp5* with heavily stained anthers and sepals, whereas in anthers, *NbrgsCaMp1::GUS* showed weak staining, and *NbrgsCaMp7::GUS* had no detectable GUS staining. Moreover, the promoter activity was obvious for *NbrgsCaMp4*, modest for *NbrgsCaMp3* and *NbrgsCaMp5*, marginal for *NbrgsCaMp1*, and undetectable for *NbrgsCaMp7* in roots (Fig. 3a). Overall, *NbrgsCaMp4* was the most active one among the *NbrgsCaMp*s (Fig. 3a), with the GUS expression level in organs increased in the order of leaf, flower and root (Fig. 3b). The other *NbrgsCaM* promoters exhibited less activity except in the leaves and flowers of *NbrgsCaMp3::GUS* when compared to their counterparts of *NbrgsCaMp4::GUS*. Noticeably, in both seedlings and mature plants, the GUS staining particularly accumulated in the veins for all of the *NbrgsCaMp::GUS* transgenic plants except *NbrgsCaMp7::GUS*, which showed no detectible expression at all (Figs. 2a and 3a).

### Analysis of *NbrgsCaMps* activity under salt and PEG treatment

**Figure 4:**
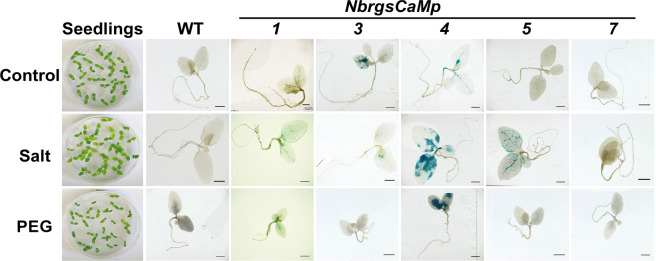
The activities of *NbrgsCaMp*s were induced under salt and PEG treatment. GUS histochemistry analysis revealed activities of *NbrgsCaMp*s under salt and PEG treatment in *NbrgsCaMp::GUS* transgenic *N. benthamiana*. The first column of plates showed that the growth status of the WT seedlings were nearly unaffected by salt treatment, but were retarded by PEG treatment. The growth status of the WT seedlings is representative of that of the other transgenic ones. The GUS expression was induced in *NbrgsCaMp1, 4* and *5* by salt treatment, and in *NbrgsCaMp1* and *4* by PEG treatment. *NbrgsCaMp4* was the most responsive one under salt and PEG treatment. Bars= 5 mm.

To investigate whether *NbrgsCaMps* respond to environmental stresses, we applied salt and PEG-simulated drought treatment to the transgenic seedlings. After transferring the seedlings to media containing additional salt or PEG for 5 days, GUS staining was carried out to detect the *NbrgsCaMps* activity. Elevated GUS staining was detected in both salt and PEG treatment for *NbrgsCaMp4::GUS* compared to the untreated ones. However, *NbrgsCaMp5::GUS* responded mainly to salt, and *NbrgsCaMp1::GUS* responded mainly to PEG, while the other samples showed no detectable response to the application of either salt or PEG (Fig. 4).

### Analysis of *rgsCaMps* activity after viral infection

**Figure 5:**
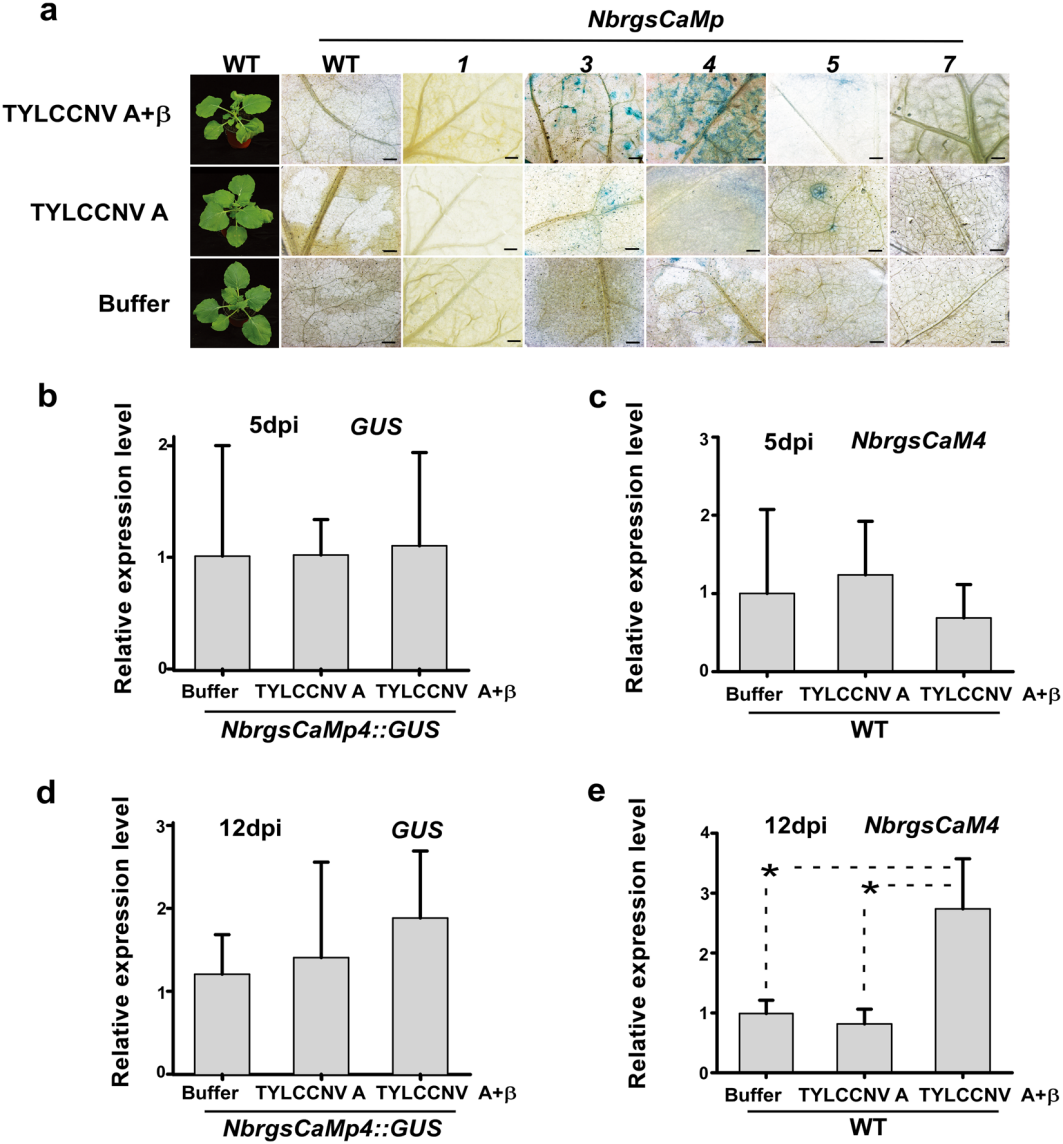
The activities of *NbrgsCaMp*s were induced by TYLCCNV inoculation. (**a**) GUS histochemistry analysis revealed activities of the *NbrgsCaMp*s induced by TYLCCNV inoculation at 7 dpi in the *NbrgsCaMp::GUS* transgenic *N. benthamiana* plants. In the first column, representative viral symptoms of the inoculated *N. benthamiana* plants at 7 dpi are shown. TYLCCNV A inoculation triggered mild induction of GUS expression in *NbrgsCaMp3::GUS*, *NbrgsCaMp4::GUS* and *NbrgsCaMp5::GUS*, whereas TYLCCNV A + β inoculation triggered stronger induction of GUS expression in these plants. The GUS staining in *NbrgsCaMp4::GUS* was most responsive to TYLCCNV A + β infection. Bars = 5 mm. (**b**,**c**) RT-qPCR results of the *GUS* and *NbrgsCaM4* expression levels showed that there was no significant elevation of promoter activity for *NbrgsCaMp4* in plants with TYLCCNV A and TYLCCNV A + β inoculation at 5 dpi. (**d**) Compared to the buffer-only inoculation, no significant elevation of *GUS* expression was detected in TYLCCNV A and TYLCCNV A + β inoculated plants at 12 dpi. (**e**) Significant elevation in *NbrgsCaM4* expression was detected in WT *N. benthamiana* at 12 dpi. *P* < 0.05. Error bars indicate S.D.

**Figure 6:**
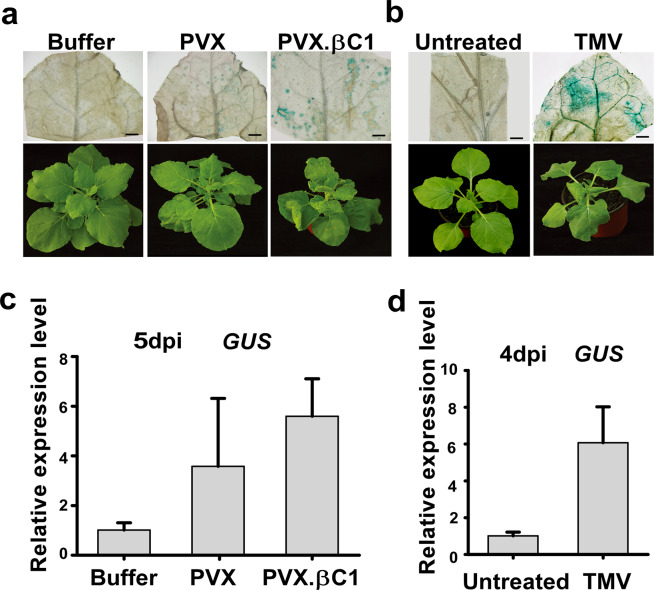
The activity of *NbrgsCaMp4* was induced by PVX and TMV inoculation. (**a**,**b**) GUS histochemistry analysis revealed activities of *NbrgsCaMp*s by PVX, PVX.βC1 and TMV inoculation, at 5 dpi and 4 dpi respectively, in *NbrgsCaMp::GUS* transgenic *N. benthamiana* with mock-inoculated or untreated plants as controls. Bars = 5 mm. (**c**,**d**) RT-qPCR results of the *GUS* expression level showed that there were significant differences in promoter activity for *NbrgsCaMp4* by TMV infection when compared to that of the controls. *P* < 0.05. Error bars indicate S.D.

To investigate the response of *NbrgsCaMps* to viral infection, we inoculated *N. benthamiana* plants with DNA A of Tomato yellow leaf curl China virus (TYLCCNV A), TYLCCNV A together with its β satellite (TYLCCNV A + β), Potato virus X (PVX), Tobacco mosaic virus (TMV), or inoculation buffer, which was used as a negative control. Then we compared the GUS staining of the systemic leaves. Because *NbrgsCaMps* activities are developmental stage-related, we carefully chose the leaves at the same developmental stage for comparison. TYLCCNV A and TYLCCNV A + β triggered induction of GUS expression in symptomatic systemic leaves of *NbrgsCaMp3::GUS*, *NbrgsCaMp4::GUS* and *NbrgsCaMp5::GUS* at 7 days post infection (dpi) (Fig. 5a). The induction difference was observed between *NbrgsCaMp4::GUS* infected by TYLCCNV A + β and by TYLCCNV A, and the TYLCCNV A + β infection induced heavier GUS staining (Fig. 5a), which was also confirmed by RT-qPCR of the expression of *NbrgsCaM4* in wild type *N. benthamiana* plants at 12 dpi (Fig. 5e). However, we failed to observe significant elevation of *GUS* expression in *NbrgsCaMp4::GUS* infected by TYLCCNV A + β comparing to TYLCCNV A and buffer only inoculation at 12 dpi (Fig. 5d). There was a difference in fold change of GUS expression between TYLCCNV A + β and buffer only inoculation, but was not as high as the previously reported case^10^. This might be due to the less sensitivity of transgene reporter and large variation between biological repeats. Nonetheless, RT-qPCR of the systemic leaves at 5 dpi (the time when viral symptoms first appear) showed that TYLCCNV A + β and TYLCCNV A triggered no difference in the expression of either *GUS* or *NbrgsCaM4* in *NbrgsCaMp4::GUS* transgenic or wild type *N. benthamiana* plants respectively (Fig. 5b,c). Additional analysis of the systemic leaves of *NbrgsCaMp4::GUS* infected by PVX or TMV showed that both viruses triggered induction of GUS expression, as evidenced by the GUS staining (Fig. 6a,b) and RT-qPCR results (Fig. 6c,d). Additionally, after inoculation with PVX.βC1 (a PVX-based viral vector for overexpression of geminivirus viral factor βC1), transient overexpression of βC1 induced higher GUS expression than by inoculation with PVX alone (Fig. 6a,c), resembling the effect of DNAβ in TYLCCNV infection (Fig. 5a,e).

Our results confirmed that inoculation with the viruses listed above (TYLCCNV A, TYLCCNV A + β, PVX, TMV, PVX.βC1), all induced GUS staining in symptomatic systemic leaves of *NbrgsCaMp4::GUS*. In both the TYLCCNV A + β-infected plants and the PVX.βC1-infected plants, the existence of βC1 triggered higher *NbrgsCaMp4* activity and *NbrgsCaM4* expressions after the initial display of symptoms. The exacerbating effect of βC1 manifested its function as a viral pathogenicity factor. However, the TYLCCNV A + β inoculated plants failed to display increase of GUS induction compared to TYLCCNV A when the early symptoms displayed at 5 dpi. It is still unclear whether the presence of βC1 induced the activation of *NbrgsCaMp4* directly or through accumulated pathogenicity of the virus.

### Analysis of *NbrgsCaMps* activity in *βC1* transgenic *N. benthamiana*

**Figure 7:**
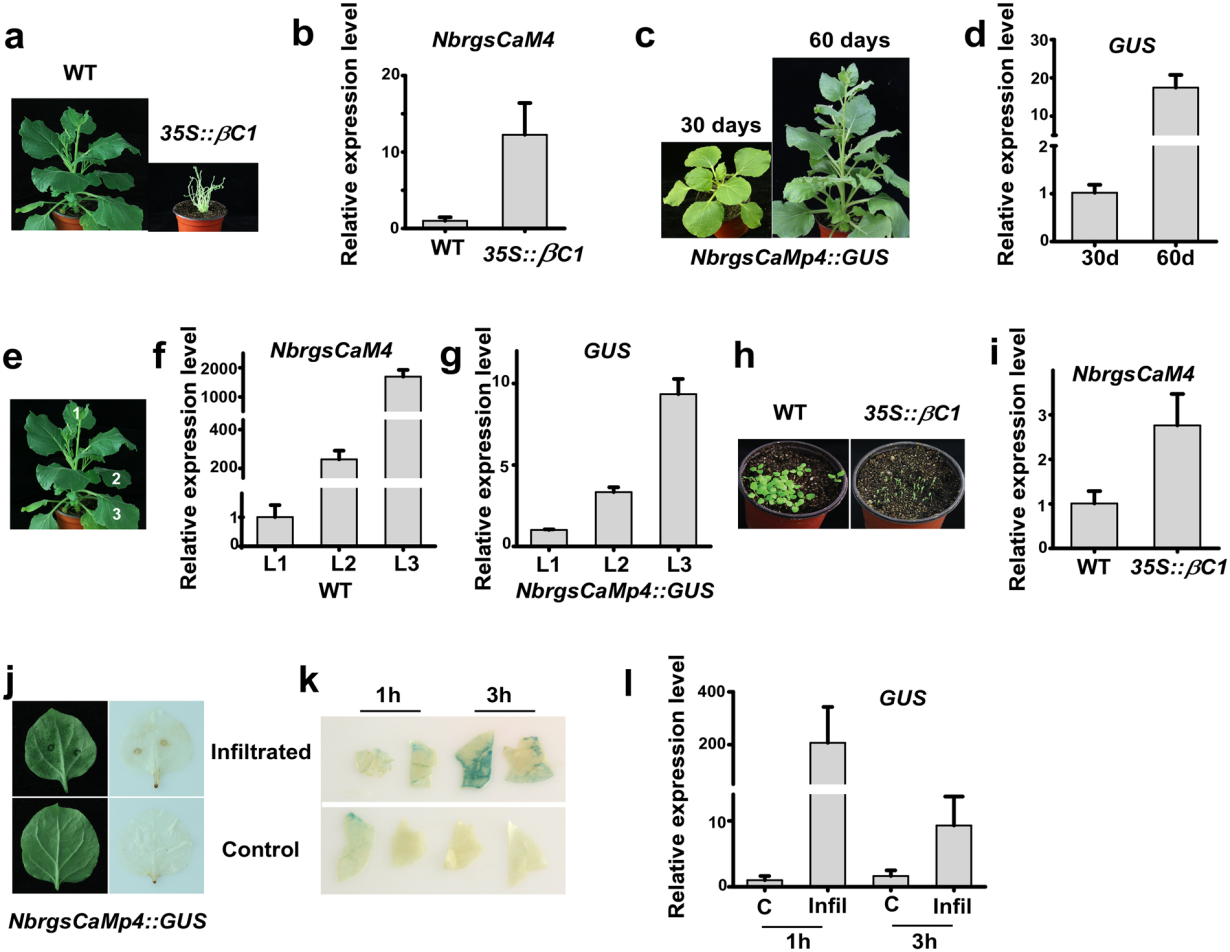
Elevated activity of *NbrgsCaMp4* during aging and under damaging treatment. (**a**) The *βC1* transgenic plants have deformed needle-like leaves. It was also severely dwarfish compared to the WT. (**b**) The *βC1* transgenic plants had higher *NbrgsCaM4* expression than WT. (**c**) The *NbrgsCaMp4::GUS* transgenic *N. benthamiana* plants at the age of 30 and 60 days post germination. (**d**) The relative *GUS* expression levels of the plants shown in c. (**e**) Leaves 1, 2 and 3 (L1, 2 and 3) are selected for the analysis of promoter activity in the WT and *NbrgsCaMp4::GUS* transgenic *N. benthamiana* plants. (**f**) The relative expression levels of *NbrgsCaMp4* in L1, 2 and 3 of the WT plants. (**g**) The relative expression levels of *GUS* in L1, 2 and 3 of the *NbrgsCaMp4::GUS* transgenic *N. benthamiana* plants. (**h**) Seedlings of the WT and *βC1* transgenic plants. (**i**) The relative expression levels of *NbrgsCaMp4* in seedlings of the WT and *βC1* transgenic plants. (**j**) Leaves of the *NbrgsCaMp4::GUS* transgenic *N. benthamiana* plants infiltrated with inoculation buffer. Left column, a leaf with infiltration wound and a leaf without treatment that served as the control. Right column, DAB staining of the infiltrated and control leaves. (**k**) GUS staining of the *NbrgsCaMp4::GUS* transgenic *N. benthamiana* leaves at 1 h and 3 h after infiltration. (**l**) The relative expression levels of *GUS* in infiltrated *NbrgsCaMp4::GUS* transgenic *N. benthamiana* leaves.

To elucidate the effect of βC1 on the activation of *NbrgsCaMp4*, we directly measured the transcription of *NbrgsCaM4* by RT-qPCR. The transcripts level of *NbrgsCaM4* was higher in *βC1* transgenic *N. benthamiana* than in WT (Fig. 7a,b). As the *βC1* transgenic *N. benthamiana* takes much longer time to grow (usually more than a year to reach the flowering stage) than the WT (50 days to reach the flowering stage), we tried to rule out the influence of leaf age on *NbrgsCaM4* expression. The results showed that, after the seedling stage, the activity of *NbrgsCaMp4* increased as the plant aged (Fig. 7c,d). Similarly, further analysis of both the *NbrgsCaM4* expression in WT and the *GUS* expression in *NbrgsCaMp4::GUS* showed that the activity of *NbrgsCaMp4* increased as the leaves aged even in the same plant (Fig. 7e–g). Thus, the influence of age on the activity of *NbrgsCaMp4* between the *βC1* transgenic and WT *N. benthamiana* becomes more important. To clarify this factor, we further tested the *NbrgsCaM4* expression in seedlings of the *βC1* transgenic and WT *N. benthamiana* at 10 days after germination (Fig. 7h). The RT-qPCR results showed that the activity of *NbrgsCaMp4* is higher in *βC1* transgenic *N. benthamiana* (Fig. 7i). However, the *βC1* transgenic *N. benthamiana* is deformed with needle-like leaves (Fig. 7a). This type of needle-like leaves mostly are composed of veins due to the pathogenicity of viral factor βC1 (Supplementary Fig. S2). As GUS staining accumulated mainly in veins of the *NbrgsCaMp::GUS* (Figs. 2a, 3a, 4 and 5a), it is difficult to exclude the influence of tissue expression bias to draw a conclusion that the up-regulation of *NbrgsCaMp4* activity is directly generated by βC1 in *βC1* transgenic *N. benthamiana*.

### Analysis of *NbrgsCaMps* activity under wounding treatment

Viral symptoms progressed as the infection persisted, and necrotic spots and yellowing usually accumulated in the infected leaves as the symptoms aggravated. For *N. benthamiana*, the infection by TYLCCNV A + β can induce higher activity of *NbrgsCaMp4* than the infection by TYLCCNV A after prolonged infection, but not at the early stage, i.e. at 12 dpi (Fig. 5e) instead of at 5 dpi (Fig. 5c). This made us wonder whether the accumulated damaging effect of TYLCCNV combined with βC1 is the direct trigger of *NbrgsCaMp4* activity, rather than the expression of βC1 itself. To investigate the damaging effect on *NbrgsCaMp4* activity, we infiltrated the inoculation buffer to the leaves of *NbrgsCaMp4::GUS* transgenic *N. benthamiana* plants to impose mechanical damage without introducing viral infection. DAB staining carried out at 1 h after infiltration revealed dark spots around the injection sites, indicating the presence of damages (Fig. 7j). RT-qPCR results showed that the transcription of *GUS* in buffer-only-infiltrated leaves elevated up to 200 fold, compared to that of the untreated control at 1 h post infiltration (Fig. 7l). This transcription elevation subsided quickly. At 3 h post infiltration, it was still about 10 fold higher than the control but not that dramatic (Fig. 7l). On the other hand, in the GUS staining results, the leaves were stained more heavily at 3 h than at 1 h, a little postponed, which might be due to the accumulation of GUS expression (Fig. 7k). These results indicated that *NbrgsCaMp4* responded instantly to damage stress. As viral infection can afflict damages to plants, the up-regulation of *NbrgsCaMp4* activity can be attributed to the side effect of viral symptoms.

## Discussion

### *NbrgsCaM*s form a unique branch of *CML* subfamily with multiple members

While *rgsCaM* was first discovered in *Nicotiana*^9^, and had been assigned complicated functions in viral responses^7,9–12^, it has been considered the only *rgsCaM* in *Nicotiana* for about 20 years. Its close homologs have been left in the shadows until this study, in which our analysis revealed 5 coding genes and 2 possible pseudogenes in *N. benthamiana* (Supplementary Data S1–S3). We named these coding genes and pseudogenes *NbrgsCaM1-7*, sequentially according to the scaffold serial numbers they reside in. *NbrgsCaM2* and *NbrgsCaM6* are counted as pseudogenes because their ORFs show no similarity to other proteins except the CaM family, and even their longest ORFs contain only an incomplete CaM function motif. Coding sequences of *NbrgsCaM1*, *3*, *4*, *5* and *7* share identities ranging from 70.4% to 93.3% (amino acid sequences identity ranging from 60.2% to 83.2%) (Supplementary Data S2 and S3). Like AtCML37, 38 and 39, the AtrgsCaMs in *Arabidopsis*^4^, NbrgsCaMs form a unique subclass of CMLs in *N. benthamiana* (Fig. 1), with close similarities to each other. In the phylogenetic tree, AtCML40 and AtCML41 coexist in the same subclass with the reported rgsCaMs (Fig. 1c). These two genes have not yet been thoroughly studied, as had the AtCML37, 38 and 39^5,6,8,24,25^. Further studies of them might be able to provide more clues to the role of rgsCaMs in plant development and stress responses.

### *NbrgsCaMs* display differential expression during development and under environmental stresses

There are about 50 CMLs in the *Arabidopsis* genome, answering to Ca^2+^ fluctuations generated through nearly all environmental, hormonal and developmental stimuli^3^. As a close subfamily of CMLs, not only the resemblance in amino acid sequences, but also the existence of identical enhancer motifs in the promoters of *AtCML37*, *38* and *39* correlate with their similar but distinct responses to environmental and developmental stimuli (Supplementary Table S1)^5,6,8,24,25^. Similarly, our analysis revealed that *NbrgsCaMp*s are enriched with developmental and stress specific enhancer elements, many of which also exist in the promoters of *AtrgsCaM*s, and each *NbrgsCaMp* has a specific combination of enhancer motifs (Supplementary Table S2 and S3). The disclosure of the amino acid sequences and enhancer elements of *NbrgsCaM*s indicates that they possess overlapping but non-identical regulatory functions in *N. benthamiana*. *NbrgsCaMp::GUS* reporter analysis revealed tissue- and developmental stage-specific promoter activities of *NbrgsCaM1*, *3*, *4* and *5* (Fig. 3), corroborating that *NbrgsCaM*s are important developmental regulatory factors. Furthermore, salt and PEG treatment induced elevated GUS expression in most of the *NbrgsCaMp::GUS* transgenic *N. benthamiana* (Fig. 4), demonstrating that *NbrgsCaMp*s respond to environmental stresses as well. Each of these *NbrgsCaMp*s drives a specific GUS expression pattern. In vegetative tissue, the GUS staining is most pronounced in veins of leaves and roots (Figs. 2a, 3a, 4 and 5a), corresponding to the vascular and root development-related promoter motifs: ATHB-2, AtMyb77, RAV1 and Root-specific nuclear factor enhancer elements (Supplementary Table S2). Among the *NbrgsCaMp*s, *NbrgsCaMp3* and *4* have the strongest GUS staining in both seedling and mature leaves (Figs. 2 and 3); *NbrgsCaMp4* and *5* responded more to salt stress than the other *NbrgsCaMps* did (Fig. 4); while *NbrgsCaMp4* is the strongest *NbrgsCaMp* in roots (Fig. 3a). All in all, *NbrgsCaMp4* is the most highly active promoter among the *NbrgsCaMp*s during vegetative growth, and responds actively to salt and PEG stress. In flower, *NbrgsCaMp4* is also one of the strongest *NbrgsCaMps* (Fig. 3). Thus, there is no wonder that *NbrgsCaM4* was the first *rgsCaM* discovered in *N. benthamiana* due to its predominant expression level^13^. On the other hand, although we failed to detect promoter activity in *NbrgsCaMp7* by GUS staining in the *NbrgsCaMp7::GUS* transgenic lines, the expression of other *NbrgsCaMs* are not to be neglected according to their promoter activities that were shown clearly by the GUS staining and RT-qPCR analyses, and undoubtedly they play important roles, considering that the activities of *NbrgsCaMp1*, *3*, and *5* are robust during development and under stress treatments (Figs. 2–5). The evidence of *NbrgsCaMs* expression can also be found in the RNA-seq data provided by the Sol Genomics Network (https://solgenomics.net/jbrowse_solgenomics/)^18,26^. The RNA_seq reads count can viewed by typing in the scaffold location of specific genes on JBrowse, the Sol Genomics Network. Despite the presence of gaps and assembly incompleteness in genomic region of some *NbrgsCaMs*, it is clear that RNA_seq reads for *NbrgsCaM1*, *3*, *4* and *5* are abundant^18,26^. The RNA_seq reads count for *NbrgsCaM4* is the highest, and for *NbrgsCaM7* is the lowest, which is only about 1/10 of that of the *NbrgsCaM4*.

### TYLCCNV infection induces the activity of *NbrgsCaMp3* and *NbrgsCaMp5*, in addition to *NbrgsCaMp4*

NbrgsCaM4 was the first discovered NbrgsCaM^13^. The namesake of “rgs” came from its role as a regulator of gene silencing in virus-plant interaction^9^. “rgs” is by far the pivotal role studied for *Nicotiana* rgsCaM, despite the discovery of developmental and stress-related functions for AtrgsCaMs in *Arabidopsis*^5–10,24,25^. In this study, we investigated *NbrgsCaMp*s responses to viral infection. Increased induction of GUS expression was detected in the systemic leaves of *NbrgsCaMp::GUS* for not only *NbrgsCaMp4*, but also *NbrgsCaMp3* and *5* after inoculation with TYLCCNV A and TYLCCNV A + β, compared to those of the untreated plants (Fig. 5a).

### βC1 induced the expression of *NbrgsCaM4* through a damaging side effect of its virulence

We detected elevated *NbrgsCaM4* expression in TYLCCNV A + β inoculated wild type *N. benthamiana*, when compared to those inoculated with TYLCCNV A at 12 dpi (Fig. 5e), similar to a previous report^10^. But quite unexpectedly, we found that TYLCCNV A and TYLCCNV A + β treatment induced equivalent GUS or *NbrgsCaM4* expression levels in systemic leaves of *NbrgsCaM4::GUS* and wild type *N. benthamiana* at 5 dpi (Fig. 5b,c). The time point of 5 dpi is when viral symptoms shown up in the TYLCCNV A + β inoculated systemic leaves. The initial viral symptoms of TYLCCNV A + β infection appeared in our observations as wrinkled curly leaves and bulging veins at 5dpi, demonstrating the presence of βC1 in the leaves that we analyzed at this time point. This suggested that βC1 possibly is not the direct factor for *NbrgsCaMp4* activation at the early stage of viral infection. Furthermore, the GUS expression levels were induced by RNA viruses, PVX and TMV (Fig. 6), similar to the results reported by Chung *et al*.^13^, suggesting that the induction of *NbrgsCaMp4* is rather a general response to viral infection, indiscriminate of DNA or RNA viruses. We have used untreated instead of mock-inoculated *N. benthamiana* plants as controls for TMV infection. This is the only place where the untreated *N. benthamiana* plants were used instead of mock-inoculated ones. As the damage induced activity of *NbrgsCaMp4* subsides quickly (Fig. 7l), and no induced upregulation of GUS expression has been detected in the mock-inoculated *NbrgsCaMp4::GUS* transgenic *N. benthamiana* plants after 3 dpi, we consider the untreated plants to be as sufficient a control as the mock-inoculated ones in TMV infection. In addition, the presence of βC1 in TYLCCNV A + β and PVX.βC1 both induced higher activity of *NbrgsCaM4* promoter (Figs. 5 and 6), suggesting that βC1 can promote the expression of *NbrgsCaM4* together with its natural master virus TYLCCNV A or the artificial viral vector PVX.

The application of βC1 together with its master viral DNA – TYLCCNV A, or with an RNA viral vector – PVX, introduced other viral factors that probably interfered with *NbrgsCaM4* expression. To single out βC1 for further analysis, we directly analyzed *NbrgsCaM4* transcription in transgenic *N. benthamiana* for *βC1* overexpression. Although in both seedlings and mature plants, the expression level of *NbrgsCaM4* was higher in *βC1* transgenic plants than in WT (Fig. 7a,b,h,i), we still cannot solely attribute the induction of *NbrgsCaM4* to βC1 directly, as we can’t rule out the impact of morphology changes and extended vegetative growth stage of the *βC1* transgenic plants on *NbrgsCaM4* expression. Our analysis of the expression levels of *NbrgsCaM4* and *GUS* in WT and *NbrgsCaMp4::GUS* transgenic *N. benthamiana* plants respectively, provided evidence that the *NbrgsCaM4p* activity increased greatly according to the advance of leaf aging in mature plants (Fig. 7c to g). Furthermore, the GUS staining accumulated in veins of *NbrgsCaMp4::GUS* (Figs. 2a, 3a, 4 and 5a), corroborating with the presence of vascular-specific enhancer element in *NbrgsCaMp4* (Supplementary Table S2). As the deformed leaves of *βC1* transgenic *N. benthamiana* are composed mostly of vascular tissue (Supplementary Fig. 2), and take a very long time to grow, it is hard to justify whether the elevated *NbrgsCaMp4* activity came from the skewed development caused by *βC1* transgene or from the βC1 factor directly. Thus, it is more reliable to analyze the induction of *NbrgsCaM4p* through TYLCCNV A + β treatment than in *βC1* transgenic plants.

TYLCCNV A is a mild virus that induces almost no symptoms to *N. benthamiana*^27,28^. On the other hand, βC1 is a pathogenic factor which is responsible for the severe viral symptoms generated by TYLCCNV A + β, such as curly leaves^29^. Thus, it was considered to be the trigger of many physiological changes in the infected plants. The fact that the TYLCCNV A + β and TYLCCNV A triggered divergence in *NbrgsCaM4p* activity happened several days after the appearance of viral symptoms is largely the reflection of the side effect of βC1 virulence, consistent with the appearance of yellowing in severely infected plants, the signature of damages and aging, which happened at the late stage of infection. It is also worth noticing that the TYLCCNV A + β treatment is not acute in induction of the *NbrgsCaMp4* activity, compared to the instant and dramatic elevation of *NbrgsCaMp4* activity triggered by wounding (Fig. 7j to l).

The elevation of *GUS* and *NbrgsCaM4* expression during aging and wounding is dramatic (Fig. 7c to g, and 7j to 7 l). But, the increase of their expression during viral infection is rather moderate (Fig. 5). Thus, compared to that during aging and wounding, the promoter activity of *NbrgsCaMp4* is far less robust under viral infection. As a regulatory factor, efficient responses are necessary for cascading amplification of signals to cope with environmental and developmental fluctuations of the surroundings. The moderate responses of *NbrgsCaMp4* to viral infection suggest that the inflictions from viral factors are not the major situations that *NbrgsCaM4* evolved to cope with, which means that probably βC1 induced the expression of *NbrgsCaM4* indirectly through damages to plants by aggravating viral symptoms. RNA‐interference (RNAi) has been reported as a surveillance system that protects the shoot tips from viral infection^30,31^. Since only low levels of *NbrgsCaM4* expression have been detected in the young leaves, especially for the very young leaves near shoot tips, it is less likely that NbrgsCaM4 can effectively suppress plant RNAi as viruses propagate (Fig. 7c to f). So far, the role NbrgsCaM4 in viral infection still needs further investigation.

### *NbrgsCaMp*s have overlapping expression patterns, indicating overlapping functions of NbrgsCaMs

From the non-negligible GUS staining in the *NbrgsCaMp3::GUS* and *NbrgsCaMp5::GUS* transgenic *N. benthamiana* plants, and their overlapping expression patterns that are similar to that of the *NbrgsCaMp4::GUS*, we deduced that besides *NbrgsCaM4*, other *NbrgsCaM*s, such as *NbrgsCaM3* and *NbrgsCaM5* probably respond redundantly to certain stimuli as a close homologous gene family. Based on our observations, though we did encounter several deformed plants randomly from tissue culture during transgene process, neither transgene of 35 S promoter-driven overexpression, nor knockdown of *NbrgsCaM4*, exhibited obvious transgene-related phenotypes. The overlapping expression patterns and similar functions of the other *NbrgsCaM*s probably mitigated the changes in expression of *NbrgsCaM4* alone, so the overexpression or knockdown of *NbrgsCaM4* yielded no obvious phenotype.

In summary, the *NbrgsCaMs* form a distinct class of *CML*s (Fig. 1c). There are more than one *rgsCaM* in both *Arabidopsis* and *N. benthamiana* (Supplementary Data S3, Fig. 1)^4,8^. They respond to developmental and environmental changes, particularly salt, drought and wounding stresses, via elevated expression to meet the need of timely regulation, and have overlapping but still distinct expression atlases which have been demonstrated by their promoter activities (Figs. 2–7). *NbrgsCaM4* is the most robustly expressed *rgsCaM* in *N. benthamiana* according to the promoter activity analysis (Fig. 2–5) and RNA-seq data from the Sol Genomics Network^18,26^. Fitting its role in regulation, *NbrgsCaMp4* responds to diverse stresses, including viral infection, though its response to viral infection is mild and probably indirect through damages owing to a side effect of viral symptoms (Figs. 6 and 7). Thus, rather than being induced by a specific viral factor, *NbrgsCaM4* is a member of a *CML* subfamily that response mostly to general developmental stages and stresses. Despite the importance of *NbrgsCaM4*, other *NbrgsCaM*s also respond actively to environmental stimuli, with overlapping expression patterns, and are probably also overlapping in functions with respect to their close homology in protein sequences. The findings of this study are helpful in characterizing not only the expression patterns, but also the relative expression strength of *rgsCaM* genes, being the first step towards a future understanding of the rgsCaM family’s multiple functions.

## Materials and Methods

### Plant materials and growth conditions

Wild type (WT) and *35 S::βC1* transgenic (generated by Qiuying Yang according to the method described before^29^) *N. benthamiana* seeds were surface-sterilized with 75% ethanol and 25% bleach for 1 min and 3 min respectively, and then washed three times with sterile water. Sterile seeds were plated on Murashige and Skoog (MS) medium plus 2.0% sucrose and 0.5% phytagel. Plated seeds were placed in a greenhouse set at 24 °C, 16-h-light/8-h-dark photoperiod for germination and growth. For culturing of mature plants, seedlings were transferred to soil after 5 days on plates and placed in the same greenhouse as above. Plants were watered as required and supplemented every other week with fertilizer. Seedlings and tissues were harvested at various time points for GUS staining and RT-qPCR analysis.

### Construction of alignments and trees

Sequences of CaM and CML proteins were downloaded from the Arabidopsis Information Resource (TAIR) (http://www.arabidopsis.org) and the Sol Genomics Network (https://solgenomics.net/organism/Nicotiana_benthamiana/genome) and subjected to phylogenetic analysis. All of the *A. thaliana* CaMs and CMLs have been listed in the previous publication^4^, except for CML51 (At1g73440), which was added in this study. While the NbCaMs and NbCMLs were obtained by blasting the Sol Genomics Network data with *A. thaliana* CaMs. Alignments were constructed using the alignment mode of ClustalW in MEGA X^32^. Note: calcineurin B-like calcium sensor proteins and calmodulin-domain protein kinases are not included as CMLs in the analyses. Multiple alignment of protein sequences was carried out with the following alignment parameters: gap opening penalty of 10, gap extension penalty of 0.2, negative matrix off and delay divergent cutoff of 30%. Protein trees were constructed using the maximum likelihood method with bootstrap of 500 embedded in the MEGA X software. Parameters for multiple alignment of nucleotide sequences were: gap opening penalty of 15, gap extension penalty of 6.66, DNA weight matrix IUB, transition weight 0.5, negative matrix off and delay divergent cutoff of 30%.

### Generation of NbrgsCaMp::GUS transgenic N. benthamiana

The promoter sequences of *NbrgsCaM*s have not been reported yet. We took 1kbp before the start codon of these genes as the putative promoters, as most of the *NbrgsCaMs* promoter elements locate within this region according to the online prediction results in this study. We cloned and constructed the promoter sequences into pBI101.GUS to generate pBI101.NbrgsCaMpn::GUS (n = 1, 3, 4, 5, 7, order of the *NbrgsCaM* homologs). Primers used in the construction are listed (Supplementary Table S4). The plant transgene was done by the Bio-run company (http://www.biorun.com/). More than 10 T0 positive transgenic lines were tested for each *NbrgsCaMpn::GUS* transgene, and homozygous T1 lines were obtained through self-fertilization of T0 plants.

### Stress treatments: salt and PEG

Salt and drought-simulation (using PEG) treatments of the *NbrgsCaMp::GUS* transgenic *N. benthamiana* plants were conducted using 5-day-old seedlings. For each stress treatment, seedlings were carefully removed from the MS plates and transferred to plates supplemented with stress reagents, and grown for an additional 5 days. Salt stress plates were simply the MS plates supplemented with 200 mM NaCl; drought simulation plates were prepared by equilibrating the MS plates with 20% PEG8000 solution (filter sterilized) overnight^33,34^. At least three independent transgenic lines of each *NbrgsCaMp::GUS* were analyzed. Samples were collected after the 5 days salt or PEG treatments for GUS staining and RT-qPCR analysis.

### Stress treatments: viral infection and wounding

For viral infection, *N. benthamiana* was grown for 4 weeks after transferring to soil to get ready for viral inoculation. Leaves were agro-inoculated with TYLCCNV A^29^, TYLCCNV A + β^29^, PVX ^35,36^, PVX.βC1^37^, TMV^38^ or mock-inoculated with inoculation buffer as described^39^. Systemic leaves from the infected plants, and equivalent leaves from the mock-inoculated ones were harvested for GUS staining and RT-qPCR analysis.

Infiltration of inoculation buffer into the mock-inoculated local leaves can cause mechanical wounding. Local leaves were harvested at specific time points for 3,3’-diaminobenzidine (DAB) staining to detect hydrogen peroxide^40^, the signal of damage generated after wounding.

### RNA extraction and RT-qPCR analysis

Total RNA was isolated using TRIzol method (Invitrogen). RNA concentration and quality were determined by spectrophotometry and gel electrophoresis. For RT-qPCR analysis, total RNA was treated with DNase I (Takara) and reverse transcribed according to the manufacturer’s instructions (EasyScript cDNA Synthesis SuperMix kit, TransGen Biotech). Specific primer pairs for *NbrgsCaM4*, *GUS* and *GAPDH* (an internal control) were listed in Supplementary Table S4. qPCR was performed using Roche LightCycler 96 with TransStart Green qPCR SuperMix (TransGen Biotech). Primer pairs were validated by cDNA template titration to ensure similar amplification kinetics and a single melting point of quantitative PCR products. Each experiment was performed in triplicate and repeated three times with different biological samples, and the results were analyzed with software supplied by the manufacturer. We used comparative CT method to determine the relative expression level of target gene expression^41^. Levels of the housekeeping gene *GAPDH* were used to calculate changes (*n*-fold) by comparing mean threshold cycle values. *P* value < 0.05 is used to delimit statistical significance. Error bars indicate S.D. To avoid genomic DNA contamination, a reaction lacking reverse transcriptase was performed in parallel for each sample.

### Histochemical assays: GUS and DAB staining

For GUS staining, leaves were immersed in GUS staining solution (GUS staining kit, HUAYUEYANG biotechnology co., LTD.) for 12 h at 37 °C in darkness, and then washed with 70% ethanol several times to remove background^42^. Samples and controls that were to be compared together were always stained in the same batch to eliminate variations caused by altering of conditions during the experiments. Stained samples were observed with Olympus SZX16 microscope (10 X amplification) and photographed by digital camera (Olympus DP72).

For DAB (3,3’-diaminobenzidine) staining, agro-infiltrated leaves were incubated in 1.0 mg/mL DAB-HCl solution in the dark overnight, then destained by boiling in 95% ethanol for 5 min. Dark brown precipitates on the leaves indicate detection of hydrogen peroxide generated after wounding^43^.

### Section of the plant leaves

Leaves of the WT and *35 S::βC1* transgenic *N. benthamiana* plants were fixed in FAA fixation buffer (containing 50% EtOH, 5% HAc and 3.7% formaldehyde) and sent to the SanShu Biotech Company (http://www.sanshubio.com) for resin embedded dissection and observation with high-resolution light microscopy according to the protocol^44^.

## Supplementary information

Supplementary information.
