# Supplementary material for: Expression patterns of NbrgsCaM family genes in Nicotiana benthamiana and their potential roles in development and stress responses
Source: Sci Rep. 2020 Jun 15;10:9652. doi: 10.1038/s41598-020-66670-x (PMC7296017; doi:10.1038/s41598-020-66670-x)
Supplement: Supplementary file 1 — Supplementary information. [file 41598_2020_66670_MOESM1_ESM.docx]

Expression patterns of *NbrgsCaM* family genes in *Nicotiana benthamiana* and their potential roles in development and stress responses

Dandan Liu^a^, and Qiuying Yang^a, b^*

^a^ State Key Laboratory for Plant Disease and Insect Pest, Institute of Plant protection, China Academy of Agricultural Sciences, Beijing 100193, China;

^b^ State Key Laboratory of Biocatalysis and Enzyme Engineering, College of Life Sciences, Hubei University, Wuhan, 430062, China.

***** Correspondence: qiuying.yang@hubu.edu.cn

**Supplementary Materials**

**Supplementary Data S1. Nucleotide sequences of *NbrgsCaM* family genes in *N. benthamiana* based on *N.benthamiana* Genome v1.0.1 Scaffolds.**

The predicted coding sequences are in upper case; other sequences are in lower case. 2kbp before the predicted start codon and 1kbp after the predicted stop codon are listed. Poly Ns are un-sequenced gaps as shown in the genome reference. “Niben101Scf…” are scaffold numbers for these sequences. The *NbrgsCaMs* are arranged in the order of their scaffold numbers.

> *NbrgsCaM1* Niben101Scf05077g00005.1

attttttgatgtcgaaggcatatagtctgagttgttgttcaatcgtagttgaacttttgggcgagggcacgtagcctaaattgtctttcaaccgtagttgaaccctttgtaattttttgatgtcgaaggcacatagcctaagttctagttcaaccgtagttgaaccctttattttcccacataacccgcttcgtgttgggcgttgggatttgttgctggtttcagatcttgcctaacctttgcttgtagtcttctcaactggttcgtacaggtcaccctgttacagatctcggttcgagatacttttgtgctttcctcctttttggcagtatcccgtgtgaaaggggggcgaggtaccttatgcgcatttttcccatagggatgggagatctacttcttcttgctcttcttccccttgctaggagacgcgggatgtggaagcaagctcgtctcgaggcttttgacttcctttcatccgacatgcctctttggcttgttcgagctgggttattttctttccttttttggtctaaggataaactatgcgattctgctataaaatctccacagacggcgccaaattatttagctcaaagttcttatacactttataagcataaatcaattaaaatcaacgaagggccaatcatagcagatcgtaataaatcaaaagaccaaataaataaaatgtaggagatagtagttattggttaaggggaggagggcaaagatcttattaatgatatcgatttggtctgcctctccatggagaggttcagagccttacaatgatgtacattcacttctttagaattggaggtaaagaagaagaagcagtccccctttagggttcttctcttcatatttatagctgtgaatcttgccagctggcgagggtcgaccgtggccgcatcgaagggaagtccgtgtacagtgagtaggtcgtagtctcgctaattaggttgagcaaccttggagtactttagccagtgaatggttatacattttattagtctgaacaagcttagaagctagttagacgcgctacttattacgattttaattctgaccaatataattacttattttgaaccggaaggagtttacctataaaactaaacgctaagaccagtaaactgtaaaccgtttgttacctctacaatatgtcacggctcgaaattcttcaaccgcatctcaccccctgaaccaaaagaaaggtaagaggagttgctaattaacaaacaagaaacaatatgccaaaaatagcttttagttcagcgccgccaatgccaaaattttattattatataaaagtagtcggaggggcagattcagaacgtaaattttataaaatttttaatattaaatttattatattataaaattatgaatttaaatctattatttattataattttaataaattttactgacacgtattaaaaatttatacttcacattaaaaattatgtattcagttgaacccgacatgtatgccctgtacaccccttaaagtagtaaaggaaacatgaaaaagaagagatgcatggcaaattcaaataagacaatattctgtatttaaaagtcaatattatatactcagtttgccttcaagataagagttatcctaaaagttacctctttctcacatcattgtcatctttcatagcgtgtgtgcttttacttttcaaacttctaagaacctatttttgtattttaatttctaaattctatacgtattaagcccacggaaaataccgacttttgaaccgccacttagttccgactaattaactaccacttcctgctccccttcctcactcatcatatataaatatcccattcttcttccttcatccccatatcctttgttgtttttcaacagctttttcaccaaaattttcccaagtttcaagttcaacccttttcgcacttaatcatattttacttatatctcaaacttccttggaaaaaaaggtagtctgaacgcgtttgttagcATGAGTATGAAGTCAGTTTCTGTACCTAGTCATGAAAGCAAGTCTGTTTTCTCAAGACTACGAAATAGGTTTTCGCTCAAAAAGGCGACTCCTCAGAAGGAAGACAAGGGTCTAACGACAACGACAACAACTGCTACAACTGACCATCTTTCCGTGAGTAGTAGTGGTAGTGAGAATAGCGAGTTAGATAGGGTATTTACGTACTTTGACGAGAACGGAGATGGAAAAGTGTCACCGACGGAGCTAAGGAGGTGTGTGAAGGCGGTAGGAGGCGAACTGACGATGGAGGAGGTGGAGATGGCAGTGAGGCTATCGGATTCTGATGGGGATGGATTGTTGGGATTGGAGGACTTTACGAAGCTAATGGAAGGAATGGAAGAAGAGAGGAATAAGGAGAGTGAGTTGATAGGAGCATTTGGAATGTATGAAATGGAnnnnnnnnnnnnnnnnnnnnnnnnnnnnnnnnnnnnnnnnnnnnnnnnnnnnnnnnnnnnnnnnnnnnnnnnnnnnnnnnnnnnnnnnnnnnnnnnnnnnnnnnnnnnnnnnnnnnnnnnnnnnnnnnnnnnnnnnnnnnnnnnnnnnnnnnnnnnnnnnnnnnnnnnnnnnnnnnnnnnnnnnnnnnnnnnnnnnnnnnnnnnnnnnnnnnnnnnnnnnnnnnnnnnnnnnnnnnnnnnnnnnnnnnnnnnnnnnnnnnnnnnnnnnnnnnnnnnnnnnnnnnnnnnnnnnnnnnnnnnnnnnnnnnnnnnnnnnnnnnnnnnnnnnnnnnnnnnnnnnnnnnnnnnnnnnnnnnnnnnnnnnnnnnnnnnnnnnnnnnnnnnnnnnnnnnnnnnnnnnnnnnnnnnnnnnnnnnnnnnnnnnnnnnnnnnnnnnnnnnnnnnnnnnnnnnnnnnnnnnnnnnnnnnnnnnnnnnnnnnnnnnnnnnnnnnnnnnnnnnnnnnnnnnnnnnnnnnnnnnnnnnnnnnnnnnnnnnnnnnnnnnnnnnnnnnnnnnnnnnnnnnnnnnnnnnnnnnnnnnnnnnnnnnnnnnnnnnnnnnnnnnnnnnnnnnnnnnnnnnnnnnnnnnnnnnnnnnnnnnnnnnnnnnnnnnnnnnnnnnnnnnnnnnnnnnnnnnnnnnnnnnnnnnnnnnnnnnnnnnnnnnnnnnnnnnnnnnnnnnnnnnnnnnnnnnnnnnnnnnnnnnnnnnnnnnnnnnnnnnnnnnnnnnnnnnnnnnnnnnnnnnnnnnnnnnnnnnnnnnnnnnnnnnnnnnnnnnnnnnnnnnnnnnnnnnnnnnnnnnnnnnnnnnnnnnnnnnnnnnnnnnnnnnnnnnnnnnnnnnnnnnnnnnnnnnnnnnnnnnnnnnnnnnnnnnnnnnnnnnnnnnnnnnnnnnnnnnnnnnnnnnnnnnnnnnnnnnnnnnnnnnnnnnnnnnnnnnnnnnnnnnnnnnnnnnnnnnnnnnnnnnnnnnnnnnnnnnnnnnnnnnnnnnnnnnnnnnnnnnnnnnnnnnnnnnnnnnnnnnnnnnnnnnnnnnnnnnnnnnnnnnnnnnnnnnnnnnnnnnnnnnnnnnnnnnnnnnnnnnnnnnnnnnnnnnnnnnnnnnnnnnnnnnnnnnnnnnnnnnnnnnnnnnnnnnnnnnnnnnnnnnnnnnnnnnnnnnnnnnnnnnnnnnnnnnnnnnnnnnnnnnnnnnnnnnnnnnnnnnnnnnnnnnnnnnnnnnnnnnnnnnnnnnnnnnnnnnnnnnnnnnnnnnnnnnnnnnnnnnnnnnnnnnnnnnnnnnnnnnnnnnnnnnnnnnnnnnnnnnnnnnnnnnnnnnnnnnnnnnnnnnnnnnnnnnnnnnnnnnnnnnnnnnnCAACTGCTACAACTGACCATCTTTCCGTGAGTAGTAGTGGTAGTGAGAATAGCGAGTTAGATAGGGTATTTACGTACTTTGACGAGAACGGAGATGGAAAAGTGTCACCGACGGAGCTAAGGAGGTGTGTGAAGGCGGTAGGAGGCGAACTGACGATGGAGGAGGTGGAGATGGCAGTGAGGCTATCGGATTCTGATGGGGATGGATTGTTGGGATTGGAGGACTTTACGAAGCTAATGGAAGGAATGGAAGAAGAGAGGAATAAGGAGAGTGAGTTGATAGGAGCATTTGGAATGTATGAAATGGAAGGGAGTGGCTACATTACTCCTAAGAGCTTGAAGAGGATGCTGAGTCGACTCAGTGAGTCAACTACCATTGATAACTGCAAAGCTATGATTCAAAGGTTTGATCTTAACGGAGACGGAGTACTCAGCTTTGACGAGTTCAAAGTTATGAtgacaagttaacaagagttcaaactacaactagtgttcgatactatataacaacaacctattataatcccacttaataaaatctaggaaggatattgtatacgcagattttatcctaccttgagagaatttatttccaaatagaccccgatattctttccatcaagaactttccatcttacttttggggatattcggactcacaacctctcaattaaaaatgaaaattgcttaccaccggagcttacgatcaaagcaaccgtcttgctctatactatttatgtatataaatattcattcgttttgggtcatgtttttgttgattcattccttctaagcaataatatgactcgagctcaaatattaattctgatatatgttttctatttagacttttcaatctgtaaattcataattgtaaagtatttcttggttacgtacgtataacttatgatgccaaggggactccagctgtaatttgtattatccacatctcgaagttttctattttcaatactttccactgttgcgtgatactctcggttcacttttaattgtcctgatattttaaaaataaattcacttttacttgttacttttagcatatcaagagaagataatttgttcttttatattatactaacagtattaattactcatatcaaattattttctgaaaattatatacattaattaatatgagtatcataataagttatgtactttatttattattttttaagaggtatgcaaagtccataataaacaagtaaaagttaacgggtttaaggtttcacacgcgctagaagtgaatgcaggataaaacttttatgggaactgctgctagcttaagaagactataagaaagaagtaaatacttaccactattcgattgtgtttacactaatgcaatgaatataggcgaaatagtatattgaccacttaaacttgtactcgtttataaaactaaaatataaatttacagtttttttatttgaacgttccaacttga

> *NbrgsCaM2* Niben101Scf05077g00027.1

agaatgtacaattaatgacatacattgttgcacttgatctcttcaccttgaactggaacctctcacgtacagccaagttcttcatcacattcataagtgtgtctttatccttataaacttgattctcttcaacaaattcgttcaacatattatcaattatacccatgttttcaccaaatttcgtgttttcaatcatttcaatatcaataatgtcagtgctatcagttgttgatacacctatagaatttgaatttgtagcaaccaaacaactggaacttgaagcaaccacagaacttgtaataaccaaatgaatcataaatctccttcactgtcacaaacaggggatattcagtgaaattcaagtttgtttctttaattctatatacaccttaactcatgtatcgttgtagatcgtgatcggtggcaaactaacatttggcacaaagttaatctctattgcgtttaaatcttcatcgatcctaatctattccgaagtagctgcaactaaattgttatagttgaatgtggactcaattattacacaatcactgatgaaattcacaaacttgttttcttcccattcatcattgtgaagaaggtaaataggaaaaatctccatataacaaaaaattgaaatcaaaattgcagcaaaaaggagaattgcgctgcttcgtattaaaaaaaattgaataaattgtagatgaagaataaatgctagggttttcgtacctgcatttattgcaagtctcgtttagaagtattttgagaagaagattggaaagaatctgagaatccagctgaatgagagaaaatcgcgcttgataatctcaaaatatttagacaagaggggttgcactggtggtaagcaacccccacttccaaccaagaggttgtgagttcgagtctccccaagagcaaggcgagaagttcttggagggaaggatgtcgggggtctatttggaaacagcctctctaccacagggtaggggtaaagtctgcgtacacactaccctccccagaccccactaagtgggattatactgggttgttgttgctgttgttgataatctcaaaataccggaaataacgcccctttttcagatttgggcctctaattgtatgctattgatttgtaaagttaaatatgggctataaaattgaaagggaggcacccaattgttttaatgtaaaatttgcccaaaactaaatgcagagaccattaaacatttcgttacacagaagacatgtcatagttcaaaattcttctacttcacctcactccttagaccaaaacgaaaggtagaaaggtaattactataacaaacaagaaacaatatgcgaaatgtagtttttaggtttaatttacagaacaaaaacacaaaccatagttcagccccacttacgccaaaatcttattttttttattaaaagaaaaggtaagaaacatgggaaagaagatatgcatgtcaaattcaaaaacgacattagaagatatagatattatataacactgtttgccttcaagataagacttatcccaaagcttggctatttctcccatcattgtcatctttcatagtgtgccttttttacttttcaaacttctaagaaccgatttttgtatttttctagaaaacaaaacgccgtacaagtttctataccgcggaaaataccgacttttggaccgccattaagtaattgccactaactaccactttcccaataaatatcccattattcttccttcatatattcatatccttggttgttttaagcgctttcaccaaaaagtttcccaagttttttttgtttgtttaagttcaaccctttttacacctgaacattcatattctacttacatctcaaactatcttgaaagaaagactagtctaaacgtggctgttagcATGTGTACTATTATGGAATCAGTTTCTGTACCTAGTGCTGAAAATAAGTCTGTTTTCTTAAGATTAAGGAAGAGGCTTTCGCTCAAAagnnnnnnnnnnnnnnnnnnnnnnnnnnnnnnnnnnnnnnnnnnnnnnnnnnnnnnnnnnnnnnnnnnnnnnnnnnnnnnnnnnnnnnnnnnnnnnnnnnnnnnnnnnnnnnnnnnnnnnnnnnnnnnnnnnnnnnnnnnnnnnnnnnnnnnnnnnnnnnnnnnnnnnnnnnnnnnnnnnnnnnnnnnnnnnnnnnnnnnnnnnnnnnnnnnnnnnnnnnnnnnnnnnnnnnnnnnnnnnnnnnnnnnnnnnnnnnnnnnnnnnnnnnnnnnnnnnnnnnnnnnnnnnnnnnnnnnnnnnnnnnnnnnnnnnnnnnnnnnnnnnnnnnnnnnnnnnnnnnnnnnnnnnnnnnnnnnnnnnnnnnnnnnnnnnnnnnnnnnnnnnnnnnnnnnnnnnnnnnnnnnnnnnnnnnnnnnnnnnnnnnnnnnnnnnnnnnnnnnnnnnnnnnnnnnnnnnnnnnnnnnnnnnnnnnnnnnnnnnnnnnnnnnnnnnnnnnnnnnnnnnnnnnnnnnnnnnnnnnnnnnnnnnnnnnnnnnnnnnnnnnnnnnnnnnnnnnnnnnnnnnnnnnnnnnnnnnnnnnnnnnnnnnnnnnnnnnnnnnnnnnnnnnnnnnnnnnnnnnnnnnnnnnnnnnnnnnnnnnnnnnnnnnnnnnnnnnnnnnnnnnnnnnnnnnnnnnnnnnnnnnnnnnnnnnnnnnnnnnnnnnnnnnnnnnnnnnnnnnnnnnnnnnnnnnnnnnnnnnnnnnnnnnnnnnnnnnnnnnnnnnnnnnnnncaactgctacaactgaccatctttccgtgagtagtagtggtagtgagaatagcgagttagatagggtatttacgtactttgacgagaacggagatggaaaagtgtcaccgacggagctaaggaggtgtgtgaaggcggtaggaggcgaactgacgatggaggaggtggagatggcagtgaggctatcggattctgatggggatggattgttgggattggaggactttacgaagctaatggaaggaATGGAAGAAGAGAGGAATAAGGAGAGTGAGTTGATAGGAGCATTTGGAATGTATGAAATGGAGGGGAGTGGCTACATTACACCTAAGAGCTTGAAGAGGATGTTGAGTCAACTCGGTGAGTCAACTTCCATTGACAACTGCAAAACTATGATACGGAGGTTTGATCTCAACGGAGATGGAGTCCTCAGCTTCGATGAATTCAGAGTTATGATGACAAGTTAGaagagttcagaaagaaatatttgtgtacataatatgattcttgagctcaaataattctgagtaatgtttttcagtgagatttttcattttgtagattcataattgcaaagagtttcttggtttcacaatttaacttggtggtcttaattaattctactttcaacttttgctggaaaaggatgctaacttctgcctgcgtagactagaaaaagatgtctccgtctaccatggtaggctggcgttctggtcaggctgtcacaaagaaagttgcccggatataattggtgttggctatagcgagggggggtggtatcaacctcaaaatacacaaggaattttccaatacggaaaaactaatttaatggggaaaaaaaggggattcaagctttaaacagaaaacttcaaactcgaattcaattaggcatgatcaagtttcaaaaaacaacaacaatccagtaaaatcacactacgctaaccttacccctaccctaaaggagaagagaggttgtttccgaaagacgctcagctcaaaaaaaacaaaaggagacaatattagtatcagcaaagaaatcatatgaaaaacaagaacatgaaattcagaagaaagatgcaaagcaaaagcgatagctagtaaatagttccagcgctagagagtgaaatagtaagacacaatattgtcgctgactatcttaaacataaaacctaccagactggtctcgcaatggtacaaagtaaggaaagactcaaaatacctcctaacctacaaccctaatactcgacctccacatctccctatccagtgtcatgtccttggaaatctgaagactcgccatatcctgcgtgatcacctctccccataacttcttaggccgccctctacctcttctcgtgccctccacaaccagctgctcacatctccgtaccagtgcatctaggcttctcctctgaacatgtccgaatcatttgagtctcgcttcccgcatcttgtcatcaatgggagtcacatgcaccttctcccgaatatcatcattcctaattttatccatcctagtatcccgcatatccacctcagcatccttatttttgctactttcatcttccggatatgtgagttctttacaggcaacactcagccacatacatcatggctggtctaacaaccgctttatagaacttacctttgagtaacggtggcactctcttatcacacaagactccagatgctaacctccaattcatctatcctacaccaatacgatacgatgtgtaacatcctcatcgatctcccctccccctggataccggcccaaggtacttgaagctgcctctacttaggatgacctatgattcaaggctcacatccacatccacttccccggctcggtgctgaactttcactccaggtagtctgtcttcgtcctgctcagcttgaaacccttagactcaag

> *NbrgsCaM* 3 Niben101Scf05588g04009.1

nnnnnnnnnnnnnnnnnnnnnnnnnnnnnnnnnnnnnnnnnnnnnnnnnnnnnnnnnnnnnnnnnnnnnnnnnnnnnnnnnnnnnnnnnnnnnnnnnnnnnnnnnnnnnnnnnnnnnnnnnnnnnnnnnnnnnnnnnnnnnnnnnnnnnnnnnnnnnnnnnnnnnnnnnnnnnnnnnnnnnnnnnnnnnnnnnnnnnnnnnnnnnnnnnnnnnnnnnnnnnnnnnnnnnnnnnnnnnnnnnnnnnnnnnnnnnnnnnnnnnnnnnnnnnnnnnnnnnnnnnnnnnnnnnnnnnnnnnnnnnnnnnnnnnnnnnnnnnnnnnnnnnnnnnnnnnnnnnnnnnnnnnnnnnnnnnnnnnnnnnnnnnnnnnnnnnnnnnnnnnnnnnnnnnnnnnnnnnnnnnnnnnnnnnnnnnnnnnnnnnnnnnnnnnnnnnnnnnnnnnnnnnnnnnnnnnnnnnnnnnnnnnnnnnnnnnnnnnnnnnnnnnnnnnnnnnnnnnnnnnnnnnnnnnnnnnnnnnnnnnnnnnnnnnnnnnnnnnnnnnnnnnnnnnnnnnnnnnnnnnnnnnnnnnnnnnnnnnnnnnnnnnnnnnnnnnnnnnnnnnnnnnnnnnnnnnnnnnnnnnnnnnnnnnnnnnnnnnnnnnnnnnnnnnnnnnnnnnnnnnnnnnnnnnnnnnnnnnnnnnnnnnnnnnnnnnnnnnnnnnnnnnnnnnnnnnnnnnnnnnnnnnnnnnnnnnnnnnnnnnnnnnnnnnnnnnnnnnnnnnnnnnnnnnnnnnnnnnnnnnnnnnnnnnnnnnnnnnnnnnnnnnctagagggcagcaaccttgtgtaaatatcttagatattcatttaggaagattctagatacttaggaaaagtctagacatttaggtagccaactataaataggcacctaaggcattcatttggaatcaagcaagaaagcaagcaatcaagcaaagttgtaaagcatccaaagcattgtaaagctctcaagttctcttaataaaagagttctttcttccaacttctagtttccttcgccaagtttctacaagcttagtctttcttaagttctactaagcatccgggtaggctgactagctaactttggcattgaaagttcgctaggccggacagatcgtcgtgtttgagttacggtctcgtcacaacaacccccgcaaaagaaaagtgaagccctatttctactttaaagaaattctttggaaacccacaagccaactttaatttaccttggtcacacgattatcatagttctcccattccttgattattattacaaattaagtacgtaagacgataacagcttttccaaatcaagtgtgatacgtacaaatgggaaataatttaatttatatatgcatagtactgtaaagaatttttattgtatcagttgctcagaattattgtaaatataattactttctaggttaccaatctcatttataattattatagttacctagtggtgtgatataattttcacaatgtcaatatatatttaaattaaaatcaacctagaaaataaaataataggagtaacttattctacacgttaaatttcactaaattatttatattatatgatatttattattattagatatcttcacccccgcttggcctttacttttgacttttaagaagcctaaaaagagaaaaggcaacaaaattgacacatattcattactgaaacccagcacgaagcttccgtacgcgcaaatcccacggaaagccgactttaaccgccgttactactttattttttccttcccatcgctttctcagcccttaaactatttatacttgctatatataacccaattctccctcttccttcatccaataccaattcttacaaatcatttcctacttttcttggttatacacttaattaacatattctgtttctcatattacaaattagctcgtttttgttttttacgatactaattaggtcggagtttccatatagcatttgcATGGAAACTATTTCTAGCTCTACAGAAAACAAGTCCGTTTTTTCAAGATTAATGAATATATTTTCGCCCAAAAAGCCGATCATAATCAAGGACGATGAGGTTATTGATCAGACGGCCAGTACTAGTACTCTTTCTGTGAGTATAATCAATACTAGCATTGAAAATAGTGATCATTTAGAGAGGGTATTTACGTACTTTGACGAGGACGGAGATGGAAAAGTGTCGCCAGTGGAGCTACAGCGGTGCGTGAGGGCGGTGGGAGGAGAGCTGACAGAAGAGGAGGCGGAGATGGCGGTGAGGCTATCGGATTCAGACGGAGATGGGATGTTAGGGTTAGAGGATTTTAGTAAATTAATGGAAGGAAGTGACGTGGAGGAAAAGAAAAAGGAGAGTGAGTTAAGAGGAGCTTTTGAGATGTATGAAATGGAAGGAACTGGTCAAATTACTCCAAAGAGTTTGAAGAGGATGTTGAGTAGACTTGGTGAGTCTACCTCTATTGATAATTGTAAAGCTATGATTCAGAGATTTGATCTTGATGGTGATGGAGTTCTTATCTTTGATGAGTTCAAAATTATGATGAACATGGAGTTAAAAAAAATCTAGtttaaagtacaaatattctcatgccttatgcatatgtataattctttgccgtttgagctcaaaataattctggttgatggattctattgttatcaagattgttattcattacttgattgttattgcaaattaattgtttgtatgtatttgacacattctacatatatacttggaagtataaagcaatataatttgcagttgagtatgaaaaaagtgaggcagtctctttataagtacgtttgcagctccaaaatacttgtgtggaataggccagagaggtttgcttgagatttgggggatgatgtcatatcagaaggatttggtttcccacatgttatgaacttataattttaagagtagatcaataagtatagtgtgaagtaatttgtgtatagattcagatgagatgaagaagatgaaatgaagaagacagctgagattgagaacaaagagtagaacacaaaattagttaagagtaacttatgttacttcattaatgaggtatgtacatgtatttatactatgtacataagtgtgtatttgtaacaaattaactaatcgccctaataactattaagtcgttaataactgacaaagctgaaaagactattatacccttatatcatttaacgctccccctcaaactaggtggtgaaaaaatattgatcatacctagtttgaatttccaatattcatgatgtactttggttaacccttttgtaaggatatcagcaggttgatctgttgttgcaatatactttgtgatgacaagtccttgttgaattctttctcttatgaagtgacaatcaatttcaaaatgcttggttcttttatggcaaactggatttgcagttatttgtatggctgccttactatcactatagatattgataggtaattgtggctttgtatcaacccctgttattaagccatatagccagataagttcagttactgttgaggctaaacttccctattctgcttttgcagagcttcgaga

> *NbrgsCaM4* Niben101Scf11105g01005.1

gtaattgctgtttcttttcttgtgtatttgcatgtgcgcgtgatttttcctccttctccataagtttcttcccctttatttataaagccgaataagtagttatctagtcttgtgcaaaaagatttataagtgggatgtgggatagtgagcagaggaaagtgggggagtggattgtgaagagggaaaaattgggaacatggtataatgatgtgttattttttagtgtgtgtgtgtgtgtgtgtgtatatatatatannnnnnnnnnnnnnnnnnnnnnnnnnnnnnnnnnnnnnnnnnnnnnnnnnnnnnnnnnnnnnnnnnnnnnnnnnnnnnnnnnnnnnnnnnnnntatatatataagagaacccaatatttataatatagtttaaattatactaagaaaaatactactaatctaaggaatgtttaaagccagcaacatataaaaacttaatttttctttttcaatcccaaaacttagaagtgatgtaactgtttccttctttaatatgtataatttgttccccaatgttgatttagcggaagcgtctattctatttataatatatatttttctagtataatttgccaaatttttatgataggaattacctttttaaacattatgcgtatacatttttaatttttaaaattattagattagattccgcaggtagtgggaacaattaccttttgtaggagaaaccataattggcagttgtaagtagtggaatctattactctacataataggtttgaaactaatttatacttctacattaaccaattaatttaaattttaacatttaaaattgagaagttctttttgttagaaagttgatttgttttgtcttaacattacatattacatgtggcaattaagtattatgtcatttgacttaatgagaagcttttgtctttaacttttaagtatatatagataacattttgtatttaaaatccttaatttggtatggcatcatattacgtaaatatatacaggcacacacacgaccatgacaaatgctaacttttcaatattaaactaactgcacccaaggctatgattagtggtcaatggaaaataaaaaattcatcaatttttaaaaaaaaacaaaaataaaactacttctagtaattagtaatttctcccatatgtccaaattttaatggactgagttatccgatatctatactagaaggtaacaaatattgcatctaatgagttaaaatatatagggtgcaaaatatttcgggcatcatggttattaaaaaatcattattaatcctccctctgtttagtttagtcaatctattttctttttggttcattccaaaaggaataactcctttttaaatttggtaataatttatttttaacttataattctaccttaaatgagaatcttttataatcatacaaatattgtggccctcttttaaacttatttagggtcacaagttctaaaactttttattttttctttaactttatgtccaattaaacaagttctatcgtaaatgtataacttgaagaatgacctacctctccctgctctaaacacaaaaaccaaacgcgtcaaacttaccttcaagataagaccatatccacaagttctgtctcacatcattatcatcatagcgtgttggccgtttaattttcaaaccttctaaaaaccggattcctttttttttttaatatagaaattaacgtcgtacaacttccaaatacaagaggctttttcataattcctactgcccagcacgaagtttccattacgtgctttgcccacggaaatatgccaactcccactgattcttcaccccaactatatatctctcttataaatatcccttttcctcttccttcatcaaaatatccaaagttttaatttcctttttcaaccctttgtattctacttacacatctcaaactatatcttgaaaggaaaataaccagtctagacttactagtttagttgttatacgtgcaatatgtgcATGGAATCGGTTTCTGTACCTAGTGTTGAAAACAAATCTTATTTCTCAAGATTAAACAAGAGGTTTTCACTCAAAAAGGCAACGACAACAACAACTATTACTGCTGATCGTCTTTCGATGAGTTGTAGTAGTAGCAGAAGTAATAATAGTAGCGAGTTAGAGAGGGTATTTACGTACTTTGACGACAACGGAGACGGCAAGGTTTCACCAGCTGAGCTAAGAAGGTGTGTGAAGGCGGTAGGAGGCGAACTGACGGTGGAGGAGGCGGAGATGGCGGTGAGACTATCGGATTCCGACGGCGATGGATTGTTGGGTTTGGAGGATTTTACAAAGCTAATGGAAGGAATGGAAGAAGAGAGGAATAAGGAGAGTGAGTTGATAGGAGCATTTGGAATGTATGAAATGGAGGGGAGTGGCTACGTTACTCCTAAGAGCTTGAAGAGGATGCTGAGTCGACTCGGTGAGTCAACTTCCATTGATAATTGCAAAGCTATGATTCAGAGATTTGATATCAATGGAGATGGAGTTCTCAGCTTTGATGAGTTCAAAGTTATGATGACAAGTTAActagatttcaacgcagaaattaaatactgtaattgtacatgtacataattctttggccttgtgcttgttgtttgtttcttaataaattaaaagataaaattatcttgagctcagaatgattttgtaaagaatttcttggtttcatgttttctaataaagccaagggaactccagctttgtattatccaattctcaaagttttttttaaatgttggtttcagctaaaaattgcgcacttctaacttgacatactcctataaatgtatggctacagtgtatcatgtatattcatattccttattcgtagaaataacccacaaagattaaattagctgtgcgaccttttgcctatccgttatccatagcacctgctaagatatgggagtgtacagataacttcaaagatgtcaacggtatataacgtttgacatcaaataacatgaatttcctgttaggcaacgtgcattaaccaaaatcaacacggttaaggaaaaataagaggaagaagaaatacaatatataattccgagttcacaatttgtttgtgtgtccttaagaaattttaacttcctcacagttgctaaggtaatggattaaatccttccaggataaaacagaataaaccttcctacgacagtgtcaatacaagaagcaggataccaacgaactcaaaaaacggagagaatcacacttacaattttcaaggaacaaaaaagctcaagaatttttggattaatgaacttgcaaagagagggaaagttattttgtagtttctgaatttctgaatgtatagaatggaagctatgcctgaatttataggcaattgttggctttgtgaaaaggtatggtgtctgttctctgtaaacagacatctgataggaagatacgttcttttaacgttacttattaatctgggtcaactagtttaactaaataaaccgggtcaatttttgaagcccaacacagtccatttaatttcccaccattacccaattcacac

> *NbrgsCaM5* Niben101Scf13289g00008.1

catctgatttagaacctcgaacaacatcttcttttttgtggatcctcctctttcttaaattgattagttggtgttcttgttactccgtatttgacacttgttgtgtagccaatttggagaacttttgtaaccttctttttgttatagtaaaactttttgaatctttgtgatcccgtggtttttaccttcgatttgaaagattttccatgttaaaatttggtagtctttatcttctttatttttgagtttgatcttcctcgacataatagaatagtgtgagccgcatgtggggaggctcacacctacagtttttagggggatctggtcgaaaaaccagtcgacatccacccgattagagggccgctgagaaattaattgagtacaatacttctcttcaaactttatcttattctgatcgttcagatggcaatcacagggtcactgagtatatatatttaactaatttagcctgacaataaaattttaggttaaacaataaatgagtttgaattattttgatgcgcggaatttaaagaattctagtacaaataaaatgaactctatcagatgtaaggatatatgtgatacaatatactacagtaaatttaatgggagcttccatgactaattttgcagagttcaagagtatatctttaccttttcccaagaaataatgaaggattttctcttgggagaacgacgaaatttttttgaggatttttgtctaagttcatataattcctaagtaagcttaggctgatgataactttcatatttttacagttaattaaattcaggaaagtagcatcttccaaattttaaaaggtatcttcattattgagatcaattaaccgtaaaagttaaaagttgtcacttttagttacaccaagtatctcttctcctaatatccaggtagtaataagtggaaattcctaaaatcaaaattgagaaatatttcaactcattccaccaaacccaaattcctaaaatcaatacgctaataaaacgttaaagtgtatagagtaatactataatttttcaacaaaaaataaaaaacacaatagtactattttatatatgcatattatagaaattaactagacttgagtgatggaaaaaaatgtgcagaaaactaaacgcagagaccattaaacatttcgttacacagcagaatgtcatagttcaaaattcttctacctcacctccctccttagaccaaaacgaaaggtagaaaggtaagtacagtaacaaacaagaaacaatatgcgaaatgtagtttttaggtttaatttacagattaaaaacacaaaccatagttcagccccacttacgccaacattttattataaaaaaaaaggtaagaaacatgggaaagaagatatgcatgtcaaattcaaaaacgacattagatgaaatagatattatataacactgtctccccaagagaaaggtggaaagttcttggagaaaatgatgtcggggtctatttggaaacaatctctctacccaagcgtaaggataagatctacgtacacactatcctccccggacccccactaaatgggattacactgggcttcttgttgttgtttgccttcaagataagacctatcccaaaacttggctatttctcacatcattgtcatctttcatagcgtgtgcctttttagttttcaaacttctacgaacctatttttgtatttttctaggaaacaaacgccgtagtggtaagcccacggaaaataccgacttttggaccgccattaagtaattgccactaactaccactttcccaatcattctccagctcacaaactaaatgttccatataaatatcccattcttcttccttccatatactcatattcttggttttttttaagagctttcaccaaagttttttttgtttgtttcaagttcaaccctttttacaccttcataattctacttacatctcaaactctcttgaaagaaagagtagtctaaacgtggttgttagcATGTGTACTATGGAATCAGTTTCTGTACCTAGTGCTGAAAATAAGTCTGTTTTCTCAAGATTAAGGAAGATGCTTTCGTTCAAAAAGGCAACTCCTCAGAAGAAACATGAGGGTCTAACAACGACTAATACAACAACTGGTCGTCTTTCAGTGAGTAGTAGTGGTAGTGACAATGGCGAGTTTGAGAGGGTATTTATATACTTTGATGAGAATGGAGATGGAAAAGTGTCACCGGCTGAGCTTAGGAAGTGTGTCAAGGCGGTAGGAGGCGAACTGACGGTGGAGGAGGCGGAGATGGCGGTGAGGCTATCGGATTCCGATGGGGATGGATTGTTGGGATTGGAGGATTTTACGAAGTTAATGGAAGGAATGGAAGAGGAGAGGAATAAGGAGAGTGAGTTGATAGGAGCATTTGGAATGTATGAAATGGAGGGGAGTGGCTACATTACTCCTAAGAGATTGAAGAGGATGCTGAGTCAACTCGGTGAGTCAACTTCCATTGACAACTGCAAAGCTATGATACGGAGGTTTGATCTAAACGGAGACGGAGTACTCAGCTTTGACGAGTTCAAAGTTATGATGACAAGTTAAcaagagttcaaatttcaactagtgccctatactatttatgtatataaacattcattcgttttgggtcttgtttttgttgatatattccttctaagtaataatatgactcttgagctcaaatattagttctgatactcctatatgtttcctatatttagacttttcaatctgtaaatttacaattagtatttgaactcgcgcaatgcacgaataatattaaaaaatatattattataataaaataattaattgttattttgtatctataatacagtgtaaccaaaactaaaataaaataataaaattagacgaaaataattatatatgttatgaaataatataaaagacaagaacaataatgcaagaataagaacaatatagagagagaatagggagagagagaagtattcttattaatacttgggataatttacaatggggtaagacccctctatttataggaagaggnnnnnnnnnnnnnnnnnnnnnnnnnnnnnnnnnnnnnnnnnnnnnnnnnnnnnnnnnnnnnnnnnnnnnnnnnnnnnnnnnnnnnnnnnnnnnnnnnnnnnnnnnnnnnnnnnnnnnnnnnnnnnnnnnnnnnnnnnnnnnnnnnnnnnnnnnnnnnnnnnnnnnnnnnnnnnnnnnnnnnnnnnnnnnnnnnnnnnnnnnnnnnnnnnnnnnnnnnnnnnnnnnnnnnnnnnnnnnnnnnnnnnnnnnnnnnnnnnnnnnnnnnnnnnnnnnnnnnnnnnnnnnnnnnnnnnnnnnnnnnnnnnnnnnnnnnnnnnnnnnnnnnnnnnnnnnnnnnnnnnnnnnnnnnnnnnnnnnnnnnnnnnnnnnnnnnnnnnnnnnnnnnnnnnnnnnnnnnnnnnnnnnnnnnnnnnnnnnnnnnnnnnnnnnnnnnnnnnnnnnnnnnnnnnnnnnnnnnnnnnnnnnnnnnnnnnnnnnnnnnnnnnnnnnnnnnnnnnnnnnnnnnnnnnnnnnnnnnnnnnnnnnnnnnnnnnnnnnnnnnnnnnnnnnnnnnnnnnnnnnnnnnnnnnnnnnnnnnnnnnnnnnnnnnnnnnnnnnnnnnnnnnnnnnnnnnnnnnnnnnnnnnnnnnnnnnnnnnnnnnnnnnnnnnnnnnnnnnnnnnnnnnnnnnnnnnnnnnnnnnnnnnnnnnnnnnnnnnnnnnnnnnnnnnnnnnnnnnnnnnnnnnnnnnnnnnnnnnnnnnnnnnnnnnnnnnnnnnnnnnnnnnnnnnnnnnnnnnnnnnnnnnnnnnnnnnnnnnnnnnnnnnnnnnnnnnnnnnnnnnnnnnnnnnnnnnnnnnnnnnnnnnnnnnnnnnnnnnnnnnnnnnnnnnnnnnnnnnnnnnnnnnnnnnnnnnnnnnnnnagATGCTTTCGTTCAAAAAGGCAACTCCTCAGAAGAAACATGAGGGTCTAACAACGACTAATACAACAACTGGTCGTCTTTCAGTGAGTAGTAGTGGTAGTGACAATGGCGAGTTTGAGAGGGTATTTATATACTTTGATGAGAATGGAGATGGAAAAGTGTCACCGGCTGAGCTTAGGAAGTGTGTCAAGGCGGTAGGAGGCGAACTGACGGTGGAGGAGGCGGTGATGGCGGTGAGGCTATCGGATTCCGATGGGGATGGATTGTTGGGATTGGAGGATTTTACGAAGTTAATGGAAGGAATGGAAGAGGAGAGGAATAAGGAGAGTGAGTTGATAGGAGCATTTGGAATGTATGAAATGGAGGGGAGTGGCTACATTACTCCTAAGAGATTGAAGAGGATGTTGAGTCAACTCGGTGAGTCAACTTCCATTGACAACTGCAAAACTATGATACGGAGGTTTGATCTAAACGGAGATGGAGTCCTCAGCTTCGATGAATTCAGAGTTATGATGACAACTTAGaagagttcagaaagaaatatttgtgtacataatatgattagaagagttcagaaagaaatatttgtgtacataatatgattcttgagctaaaataatcctgagtaacgtttttcagtgagatttttcattttgtagattcatgattgcaaagaactcttggtttcacaatttcacaatttcacaatttaacttggtgctaattctactttcaactttcaacttttgctggaaacggatgctaacttctgcctccgtaaactagaaaaaaatgtctcccttaagctggcgttctggtcaggctgtcacaaggaaagttgcccggatataattggtgttgactatagggagggggggagggggtatcaacctcaaaatacacaaggaattgtccaataaggaaaaactaatttaatggaaaaacaaaagggagtcaaacttcaaactcgaattcaattaggcatgatcaggtttcagagaacagcactctaaatttcagatctagaaaagatcatatgcgtgaattgataggctagaactaggaaaagaggagaggtagactgatgaggctggaaattcagctactcaatgatgctaagttttgctaaggaattaaggtttgtacaaactagctacagcagatcgaggtaatcataatgaaagaaaagtctcagccagaaaaaaaaatggaagtcaaaatccagagatatactttggaaacactaaaggaaagagagattactttctttcagattgattgaacttttcacaagttattcacaatcattaataatagtttgtaatacataatggtttgctagaggagaaattacattgacaatacatgagggcagcatgcaaagagaagatgctagttgtcaattccaagcaacggattcagctcaagcactgaccttcgtatatcatgtagatcctcctctaccttgtcggaaaaagagcatgattggcatgaaaatgcattcagcaactgactttttctggt

> *NbrgsCaM* 6 Niben101Scf13289g00011.1

agaaagaatgaaaaatgaacatgtatagcgcagtattttaaaacgctgtgcatgaaaatgctggtataaaattatgttatatataaaatgatcattttttctaggaagaattacattctttattatttggtccaacaacttatttgaaaatgatctatatttgaaattcttatttaatataactcaggttgtatataatataaaaattaatttgcaatagttagcatttcttatttctcccttcccttttttacttcgttcttctttttctttccatgccagcttttgcatcataaaatttttctactactgttattttccttcaattttttttttgaattttagtggttaactaaggatttgcaaaataaaaagtcaccattacaagtggtttgaagattcgatttcagaaattgagttttctaatttgttagttgtttggaatgagtgttgtctcaattgattggaatgattaaactaagtttaaaacttaaatttcaagtgatttggagtagatttgggctatatttgagttaaattttaaaatttgagttttccgatttgttagttgtttggagtgtgtgttctcgctactgattggaatcgtcaagataagttaagaacttaaatttcaagtgatttggagtagctttaggttatatttgagttaaattttagcggatgcccaaggaagaagaagaagaacacgtgaagctccattgataggattcatttgtataatattgtataaatagtatataatagtatataaacacaacttatacactattatatccaaggttgatacatcatttacatgtatttggtgaatttctcgcattttttcttctttttctttgtcttctcgttctcctcatatacctatatacataatgtatcaaaagtatttttactctgtgattatgtatctttatacacttatatataactatatacatattgtacttctttgtataaaagtgtataatattgtataaaagtgtataaacatcgcaggcgaaatttttgtacgattttcacttacaattcttatataaaactaattcacatctccagcaaataacttcaaacttcgtatacaacttacttatactatttttaataagtttaaacaacaatcattccaaatttctcacaaaatcataatcgaaatttaacaacattagtattaaaggagatgagattttaggtttctcgcgtctgtttttgcgttttgcagttgtgataggtatgtataaacctgaagctatacaaaattttagaaaaaaatatagaagtattggttatggaagaaggaacgaatgagtgttaactggatgaggaactaatttctcttttcaattttatttgcatatgattttttttattaattaaataaaatatttataggtgaaaaataaggagaaagcaagaataaagataagtagggataaataagattattgtaactataagaagtgagaaaaaaaagatcttataaatatttgattacatatttataaacttataattaaactaaaatagtgcaatatcaatttatgaggtatattattatataattttatcttttttatatatatttatattacacttgtccaaaaagatcacaattaagttccggtctcttcgaattgccgtcaagataagactcatccaaaaagtgagctctttctcacatcaaacatagcgcgtgtacttttacttttcaaacttctaagaacctatttttgtattttaatttctagaagtttctataagtgggaaaatacagaattttggaccgccatttagttgccactaactattcccactcttcatatgtaattaaatatcccattcttcttccttcatccccataggccatatcctttgttgtttttcaacagctctttaccaaaagttttctttgtttcaacttaaaaccttttcgcattctacttacatctcaaacttccttgggtataaacgcgtttgttagcATGAGTATGCAGTCAGTTTCTGTACCTAGTCATGAAAACAAGTCTGCTTTCTCAAGACTACGCAATAGGTTTTCGCTCAGAAGGAAGACGAACGTCTAAcgacgacgacaacaactgaccatctttcggtaagtagtagtgagaatagcgagttagagagggtatttacgtactttgacgaaaacggagacggcaaggtttcaccagctgagctaagaaggtgtgtgaaggcggtaggaggcgaactgacggtggaggaggcggtgATGGCGGTGAGGCTATCGGATTCCGATGGAGATGGATTGTTGGGATTGGAGGATTTTACGAAGTTAATGGAAGGAATGGAAGAGGAGAGGAATAAGGAGAGTGAGTTGATAGGAGCATTTGGAATGTATGAAATGGAGGGGAGTGGCTACATTACTCCTAAGAGCTTGAAGAGGATGCTGAGTCAACTCGGTGAGTCAACTTCCATTGACAACTGCAAAGCTATGATACGGAGGTTTGATCTAAACGGAGACGGAGTACTCAGCTTTGACGAGTTCAAAGTTATGATGACAAGTTAAcaagagttcaaatttcaactagtgccctatactatttatgtatataaacattcattcgttttgggtcttgtttttgttgatatattccttctaagtaataatatgactcttgagctcaaatattagttctgatactcctatatgtttcctatatttagacttttcaatctgtaaatttacaattagtatttgaactcgcgcaatgcacgaataatattaaaaaatatattattataataaaataattaattgttattttgtatctataatacagtgtaaccaaaactaaaataaaataataaaattagacgaaaataattatatatgttatgaaataatataaaagacaagaacaataatgcaagaataagaacaatatagagagagaatagggagagagagaagtattcttattaatacttgggataatttacaatggggtaagacccctctatttataggaagaggnnnnnnnnnnnnnnnnnnnnnnnnnnnnnnnnnnnnnnnnnnnnnnnnnnnnnnnnnnnnnnnnnnnnnnnnnnnnnnnnnnnnnnnnnnnnnnnnnnnnnnnnnnnnnnnnnnnnnnnnnnnnnnnnnnnnnnnnnnnnnnnnnnnnnnnnnnnnnnnnnnnnnnnnnnnnnnnnnnnnnnnnnnnnnnnnnnnnnnnnnnnnnnnnnnnnnnnnnnnnnnnnnnnnnnnnnnnnnnnnnnnnnnnnnnnnnnnnnnnnnnnnnnnnnnnnnnnnnnnnnnnnnnnnnnnnnnnnnnnnnnnnnnnnnnnnnnnnnnnnnnnnnnnnnnnnnnnnnnnnnnnnnnnnnnnnnnnnnnnnnnnnnnnnnnnnnnnnnnnnnnnnnnnnnnnnnnnnnnnnnnnnnnnnnnnnnnnnnnnnnnnnnnnnnnnnnnnnnnnnnnnnnnnnnnnnnnnnnnnnnnnnnnnnnnnnnnnnnnnnnnnnnnnnnnnnnnnnnnnnnnnnnnnnnnnnnnnnnnnnnnnnnnnnnnnn

> *NbrgsCaM* 7 Niben101Scf23800g00001.1

nnnnnnnnnnnnnnnnnnnnnnnnnnnnnnnnnnnnnnnnnnnnnnnnnnnnnnnnnnnnnnnnnnnnnnntatatatatatatatatatatatatatatatatatatatatatatatatatatatatataatagttcatctcatatataaattatattgaaacaatataatttatgtttgctttaaatgtacatctgtattctgtaatataatatctttgaagttgttgctctactcataataagaatttatgctgaatacgcttgtatatttgataattctttatctttatcaggtaggtttagaagggaacttgtttaaaataaaatttgaattcaagaatattaataattcagtaagtaagaaataactggggtttaaaattacaaaacgaaagacaaaaaagcaaaagctgaagaagaaagaaaagaactggaacacacaagcactagaaagaaaagagaactaaataactttagctctagagttaaagttcgatagttacaaaataaaaagtttagctctagagctgaagttatttagttctcttttctttgtagttgatcaatcgcgaaacaccaaaaacagtagtgattgcctcagacttcagctctataactgaagtttcctagttacaacacagaaatcgctgaagtttcccaattacaacacaaaaactttagttctaaagctgaagtttaccaacaaaaatttcagcctagagctgaagcttacaaacaaaaacttgagctctagaactgaagttcgatagttacaaaaaataaacttcttctctagagctgaacttcaagcctagctactacaatattgaagttttgcgtgattacctttgctatttcagattcatatgctgcagttatgcgaaaaagcagatacgcttgtaattttttttcgcaaaacaaacataaattaaaacgtaacacaaaaacggatatagacgcaaatacccctcaatatcccaaccccgcaaaagaaaagtgaagcccgatttctacttttaaaagcttgtttgaaaagccacaacccaactttaatttaccttgttcaaccgactcgcacatgaccctctttgtttttgattgaagagttaagactccctgaataatatctcacaaggtcctggcaaaacaacgcgaaaaataagtatccgtatagggaaaaacaggcatcattctgctgtggttttcaaatcaagattgataggtacaagttggggaaaaaagttggaaatgatttaatttatatgcagaatactataagaattttatactattagtatgcccaaaattaattattgtaacaaatatacgatttctttttctaggttacgaatcttattaataattattatagttatctcttagttgacctaattaatagtgtgatataacttttacaatgtcaatatatatttagatcaaaatcaacctagaaaataaaataataggagtaacttattctactcattaactttcaccaaattttccaccaaaagaaaaactttcactaaattctttatattataaaatattattatcattaaatatcttcacacacacttgacgtttacttttgacttttaagaaccctaaaaaagaaaaggcaacaaattgatacatattcattactgaaacccagcacgaagcttccgtacgcgcgaatcccacgggaagccgacttcaaccgccattaactaacctcttccccattacgacttctttattttttccttcccatcgctttctcagcccttaaactgtttatacttgctatatataacccaattctttctcttccttcctccaataccaattgttattctcatgcaacaaccatctttcttacaaaaaattcaaaactttatttgatcaagtccaactaacccgttatacacttaataatcatattctgcttctcatattacaaattagctagttttttggttttacgttactaaggccggagtttgcatatagcATGTGCATGGATACTACTTCTAGCTCTGCAGAAAATAACAAGTCCGTTTTTTCAAGATTAAGGAATAGGTTTTCACCCAAAAAGCCGATCGTAATCAAGAATAACGATGAGGTTATTGATCAGACGGCCAGTACTAGTACTCTTTCTGTGAGTTCTGAAAATATTGATCATTTGGAGAGGGTATTTACGTACTTTGACGAGGACGGAGATGGAAAAGTCTCGCCAGCGGAGCTACAGCGCTGCGTGAGGGCGGTAGGAGGAGAGCTGACGGAGGAGGAGGCGGAGATGGCGGTGAGGCTATCGGATTCAGATGGAGATGGGATGTTAGGGTTAGAGGATATTAGTAAATTAATGGAAGGAGGTGACGTGGAGGAAAAGAATAAGGGGACTGGGTTAAGAGGAGCTTTTGAGATGTATGAAATGGAAAGAACTGGTCAAATTACTCCAAAGAGTTTGAAGAGGATGTTGAATAGACTTGGTGAGTCTACCTCTGTTGATAATTGCAAATCTATGATTCAAAGATTTGATCTTGATGGTGACGGAGCTCTCAACTTTGATGAGTTCAAAATTATGATGAACATGGAGTTAAAAAACATCTAGtttaaagtacaaatattcccatgccttatgtatatacataattcttttccttttgagctcaaaataattctggttgatggattctattgtcatcaagattgttatccattacttgattgttattgcaaattgattgtttgtatgtattggacaccattctacatatatacttggaaataaagcaatataatttgcagttgaggatgaaaaaagtgaatcaaatacatacagctccaaaatactagcgtggaataggccagagaggtttgcttgagatttgggggatgatgtcatataagaaggatttggtttcccaccttatatcatcatgttatgaactcataattttaagagtagatcataagtccgcgcttgtaacaatccagatttctcaatcttgtaaatcgtgatggcaccgattcatgaaagctagacaagtcggtattatagattccttaatctctttacttagccctttttaacagttcaccataacaggtaaaaacaacgaaaacattataataaacaaaaatgcggaagacaaaaactaatagtttaaccatatattagtgcagaatccaacatacaactctatccagaatttggtgtcacgatgtcacgaagtatctacgaatattactgtctgaaagctaaatacaaaactgtctctaaaatacatataagaaacagaatggagaaagatagtaggaaggggaccccaaggcatgcggacgcctgcaggactacctcgggtctcttgatggattgaaggcagcaacctctctctacgatccaaatgctctagcatcaggatttttacacagtgtagtgtaatatcaacacaaccgaccacatgcgctggtaagtgtctagatgtgacgacccgggcagtcgtctcatgagttaccctctgtttcccctattaatgcttcgttatccatgtttttatgatatcggattggtcggatcgaatccacaataatttgataagctttgagac

**Supplementary Data S2. Nucleotide sequences of the *NbrgsCaM* family genes verified/corrected by PCR cloning.**

The predicted coding sequences are in upper case; other sequences are in lower case.

> *NbrgsCaM* *1*

attttttgatgtcgaaggcatatagtctgagttgttgttcaatcgtagttgaacttttgggcgagggcacgtagcctaaattgtctttcaaccgtagttgaaccctttgtaattttttgatgtcgaaggcacatagcctaagttctagttcaaccgtagttgaaccctttattttcccacataacccgcttcgtgttgggcgttgggatttgttgctggtttcagatcttgcctaacctttgcttgtagtcttctcaactggttcgtacaggtcaccctgttacagatctcggttcgagatacttttgtgctttcctcctttttggcagtatcccgtgtgaaaggggggcgaggtaccttatgcgcatttttcccatagggatgggagatctacttcttcttgctcttcttccccttgctaggagacgcgggatgtggaagcaagctcgtctcgaggcttttgacttcctttcatccgacatgcctctttggcttgttcgagctgggttattttctttccttttttggtctaaggataaactatgcgattctgctataaaatctccacagacggcgccaaattatttagctcaaagttcttatacactttataagcataaatcaattaaaatcaacgaagggccaatcatagcagatcgtaataaatcaaaagaccaaataaataaaatgtaggagatagtagttattggttaaggggaggagggcaaagatcttattaatgatatcgatttggtctgcctctccatggagaggttcagagccttacaatgatgtacattcacttctttagaattggaggtaaagaagaagaagcagtccccctttagggttcttctcttcatatttatagctgtgaatcttgccagctggcgagggtcgaccgtggccgcatcgaagggaagtccgtgtacagtgagtaggtcgtagtctcgctaattaggttgagcaaccttggagtactttagccagtgaatggttatacattttattagtctgaacaagcttagaagctagttagacgcgctacttattacgattttaattctgaccaatataattacttattttgaaccggaaggagtttacctataaaactaaacgctaagaccagtaaactgtaaaccgtttgttacctctacaatatgtcacggctcgaaattcttcaaccgcatctcaccccctgaaccaaaagaaaggtaagaggagttgctaattaacaaacaagaaacaatatgccaaaaatagcttttagttcagcgccgccaatgccaaaattttattattatataaaagtagtcggaggggcagattcagaacgtaaattttataaaatttttaatattaaatttattatattataaaattatgaatttaaatctattatttattataattttaataaattttactgacacgtattaaaaatttatacttcacattaaaaattatgtattcagttgaacccgacatgtatgccctgtacaccccttaaagtagtaaaggaaacatgaaaaagaagagatgcatggcaaattcaaataagacaatattctgtatttaaaagtcaatattatatactcagtttgccttcaagataagagttatcctaaaagttacctctttctcacatcattgtcatctttcatagcgtgtgtgcttttacttttcaaacttctaagaacctatttttgtattttaatttctaaattctatacgtattaagcccacggaaaataccgacttttgaaccgccacttagttccgactaattaactaccacttcctgctccccttcctcactcatcatatataaatatcccattcttcttccttcatccccatatcctttgttgtttttcaacagctttttcaccaaaattttcccaagtttcaagttcaacccttttcgcacttaatcatattttacttatatctcaaacttccttggaaaaaaaggtagtctgaacgcgtttgttagcATGAGTATGAAGTCAGTTTCTGTACCTAGTCATGAAAGCAAGTCTGTTTTCTCAAGACTACGAAATAGGTTTTCGCTCAAAAAGGCGACTCCTCAGAAGGAAGACAAGGGTCTAACGACAACGACAACAACTGCTACAACTGACCATCTTTCCGTGAGTAGTAGTGGTAGTGAGAATAGCGAGTTAGATAGGGTATTTACGTACTTTGACGAGAACGGAGATGGAAAAGTGTCACCGACGGAGCTAAGGAGGTGTGTGAAGGCGGTAGGAGGCGAACTGACGATGGAGGAGGTGGAGATGGCAGTGAGGCTATCGGATTCTGATGGGGATGGATTGTTGGGATTGGAGGACTTTACGAAGCTAATGGAAGGAATGGAAGAAGAGAGGAATAAGGAGAGTGAGTTGATAGGAGCATTTGGAATGTATGAAATGGAAGGGAGTGGCTACATTACTCCTAAGAGCTTGAAGAGGATGCTGAGTCGACTCAGTGAGTCAACTACCATTGATAACTGCAAAGCTATGATTCAAAGGTTTGATCTTAACGGAGACGGAGTACTCAGCTTTGACGAGTTCAAAGTTATGATGACAAGTTAAcaagagttcaaactacaactagtgttcgatactatataacaacaacctattataatcccacttaataaaatctaggaaggatattgtatacgcagattttatcctaccttgagagaatttatttccaaatagaccccgatattctttccatcaagaactttccatcttacttttggggatattcggactcacaacctctcaattaaaaatgaaaattgcttaccaccggagcttacgatcaaagcaaccgtcttgctctatactatttatgtatataaatattcattcgttttgggtcatgtttttgttgattcattccttctaagcaataatatgactcgagctcaaatattaattctgatatatgttttctatttagacttttcaatctgtaaattcataattgtaaagtatttcttggttacgtacgtataacttatgatgccaaggggactccagctgtaatttgtattatccacatctcgaagttttctattttcaatactttccactgttgcgtgatactctcggttcacttttaattgtcctgatattttaaaaataaattcacttttacttgttacttttagcatatcaagagaagataatttgttcttttatattatactaacagtattaattactcatatcaaattattttctgaaaattatatacattaattaatatgagtatcataataagttatgtactttatttattattttttaagaggtatgcaaagtccataataaacaagtaaaagttaacgggtttaaggtttcacacgcgctagaagtgaatgcaggataaaacttttatgggaactgctgctagcttaagaagactataagaaagaagtaaatacttaccactattcgattgtgtttacactaatgcaatgaatataggcgaaatagtatattgaccacttaaacttgtactcgtttataaaactaaaatataaatttacagtttttttatttgaacgttccaacttga

> *NbrgsCaM* *2*

tgaagcaaccacagaacttgtaataaccaaatgaatcataaatctccttcactgtcacaaacaggggatattcagtgaaattcaagtttgtttctttaattctatatacaccttaactcatgtatcgttgtagatcgtgatcggtggcaaactaacatttggcacaaagttaatctctattgcgtttaaatcttcatcgatcctaatctattccgaagtagctgcaactaaattgttatagttgaatgtggactcaattattacacaatcactgatgaaattcacaaacttgttttcttcccattcatcattgtgaagaaggtaaataggaaaaatctccatataacaaaaaattgaaatcaaaattgcagcaaaaaggagaattgcgctgcttcgtattaaaaaaaattgaataaattgtagatgaagaataaatgctagggttttcgtacctgcatttattgcaagtctcgtttagaagtattttgagaagaagattggaaagaatctgagaatccagctgaatgagagaaaatcgcgcttgataatctcaaaatatttagacaagaggggttgcactggtggtaagcaacccccacttccaaccaagaggttgtgagttcgagtctccccaagagcaaggcgagaagttcttggagggaaggatgtcgggggtctatttggaaacagcctctctaccacagggtaggggtaaagtctgcgtacacactaccctccccagaccccactaagtgggattatactgggttgttgttgctgttgttgataatctcaaaataccggaaataacgcccctttttcagatttgggcctctaattgtatgctattgatttgtaaagttaaatatgggctataaaattgaaagggaggcacccaattgttttaatgtaaaatttgcccaaaactaaatgcagagaccattaaacatttcgttacacagaagacatgtcatagttcaaaattcttctacttcacctcactccttagaccaaaacgaaaggtagaaaggtaattactataacaaacaagaaacaatatgcgaaatgtagtttttaggtttaatttacagaacaaaaacacaaaccatagttcagccccacttacgccaaaatcttattttttttattaaaagaaaaggtaagaaacatgggaaagaagatatgcatgtcaaattcaaaaacgacattagaagatatagatattatataacactgtttgccttcaagataagacttatcccaaagcttggctatttctcccatcattgtcatctttcatagtgtgccttttttacttttcaaacttctaagaaccgatttttgtatttttctagaaaacaaaacgccgtacaagtttctataccgcggaaaataccgacttttggaccgccattaagtaattgccactaactaccactttcccaataaatatcccattattcttccttcatatattcatatccttggttgttttaagcgctttcaccaaaaagtttcccaagttttttttgtttgtttaagttcaaccctttttacacctgaacattcatattctacttacatctcaaactatcttgaaagaaagactagtctaaacgtggctgttagcATGTGTACTATTATGGAATCAGTTTCTGTACCTAGTGCTGAAAATAAGTCTGTTTTCTTAAGATTAAGGAAGAGGCTTTCGCTCAAAaggcaactcctcagaaagaagatgagggtctaacaacgaatactacaacaactggtcgcctttcggtgagtagtagtgatagtgacaatgatgagttagaaagggtatttacgtactttgacgagatgcagacggcaaggtttcaccggcggagctaaggaggtgcgtgaaggcggtaggaggcgagttgaaggtggatgaggcggagatggcagtgaggctatcggattctgatggggatggattgttgggattggaggactttacgaagctaatggaaggaATGGAAGAAGAGAGGAATAAGGAGAGTGAGTTGATAGGAGCATTTGGAATGTATGAAATGGAGGGGAGTGGCTACATTACACCTAAGAGCTTGAAGAGGATGTTGAGTCAACTCGGTGAGTCAACTTCCATTGACAACTGCAAAACTATGATACGGAGGTTTGATCTCAACGGAGATGGAGTCCTCAGCTTCGATGAATTCAGAGTTATGATGACAAGTTAGaagagttcagaaagaaatatttgtgtacataatatgattcttgagctcaaataattctgagtaatgtttttcagtgagatttttcattttgtagattcataattgcaaagagtttcttggtttcacaatttaacttggtggtcttaattaattctactttcaacttttgctggaaaaggatgctaacttctgcctgcgtagactagaaaaagatgtctccgtctaccatggtaggctggcgttctggtcaggctgtcacaaagaaagttgcccggatataattggtgttggctatagcgagggggggtggtatcaacctcaaaatacacaaggaattttccaatacggaaaaactaatttaatggggaaaaaaaggggattcaagctttaaacagaaaacttcaaactcgaattcaattaggcatgatcaagtttcaaaaaacaacaacaatccagtaaaatcacactacgctaaccttacccctaccctaaaggagaagagaggttgtttccgaaagacgctcagctcaaaaaaaacaaaaggagacaatattagtatcagcaaagaaatcatatgaaaaacaagaacatgaaattcagaagaaagatgcaaagcaaaagcgatagctagtaaatagttccagcgctagagagtgaaatagtaagacacaatattgtcgctgactatcttaaacataaaacctaccagactggtctcgcaatggtacaaagtaaggaaagactcaaaatacctcctaacctacaaccctaatactcgacctccacatctccctatccagtgtcatgtccttggaaatctgaagactcgccatatcctgcgtgatcacctctccccataacttcttaggccgccctctacctcttctcgtgccctccacaaccagctgctcacatctccgtaccagtgcatctaggcttctcctctga

> *NbrgsCaM* *3*

Same as the published one

> *NbrgsCaM* *4*

Same as the published one

> *NbrgsCaM* *5*

catctgatttagaacctcgaacaacatcttcttttttgtggatcctcctctttcttaaattgattagttggtgttcttgttactccgtatttgacacttgttgtgtagccaatttggagaacttttgtaaccttctttttgttatagtaaaactttttgaatctttgtgatcccgtggtttttaccttcgatttgaaagattttccatgttaaaatttggtagtctttatcttctttatttttgagtttgatcttcctcgacataatagaatagtgtgagccgcatgtggggaggctcacacctacagtttttagggggatctggtcgaaaaaccagtcgacatccacccgattagagggccgctgagaaattaattgagtacaatacttctcttcaaactttatcttattctgatcgttcagatggcaatcacagggtcactgagtatatatatttaactaatttagcctgacaataaaattttaggttaaacaataaatgagtttgaattattttgatgcgcggaatttaaagaattctagtacaaataaaatgaactctatcagatgtaaggatatatgtgatacaatatactacagtaaatttaatgggagcttccatgactaattttgcagagttcaagagtatatctttaccttttcccaagaaataatgaaggattttctcttgggagaacgacgaaatttttttgaggatttttgtctaagttcatataattcctaagtaagcttaggctgatgataactttcatatttttacagttaattaaattcaggaaagtagcatcttccaaattttaaaaggtatcttcattattgagatcaattaaccgtaaaagttaaaagttgtcacttttagttacaccaagtatctcttctcctaatatccaggtagtaataagtggaaattcctaaaatcaaaattgagaaatatttcaactcattccaccaaacccaaattcctaaaatcaatacgctaataaaacgttaaagtgtatagagtaatactataatttttcaacaaaaaataaaaaacacaatagtactattttatatatgcatattatagaaattaactagacttgagtgatggaaaaaaatgtgcagaaaactaaacgcagagaccattaaacatttcgttacacagcagaatgtcatagttcaaaattcttctacctcacctccctccttagaccaaaacgaaaggtagaaaggtaagtacagtaacaaacaagaaacaatatgcgaaatgtagtttttaggtttaatttacagattaaaaacacaaaccatagttcagccccacttacgccaacattttattataaaaaaaaaggtaagaaacatgggaaagaagatatgcatgtcaaattcaaaaacgacattagatgaaatagatattatataacactgtctccccaagagaaaggtggaaagttcttggagaaaatgatgtcggggtctatttggaaacaatctctctacccaagcgtaaggataagatctacgtacacactatcctccccggacccccactaaatgggattacactgggcttcttgttgttgtttgccttcaagataagacctatcccaaaacttggctatttctcacatcattgtcatctttcatagcgtgtgcctttttagttttcaaacttctacgaacctatttttgtatttttctaggaaacaaacgccgtagtggtaagcccacggaaaataccgacttttggaccgccattaagtaattgccactaactaccactttcccaatcattctccagctcacaaactaaatgttccatataaatatcccattcttcttccttccatatactcatattcttggttttttttaagagctttcaccaaagttttttttgtttgtttcaagttcaaccctttttacaccttcataattctacttacatctcaaactctcttgaaagaaagagtagtctaaacgtggttgttagcATGTGTACTATGGAATCAGTTTCTGTACCTAGTGCTGAAAATAAGTCTGTTTTCTCAAGATTAAGGAAGATGCTTTCGTTCAAAAAGGCAACTCCTCAGAAGAAACATGAGGGTCTAACAACGACTAATACAACAACTGGTCGTCTTTCAGTGAGTAGTAGTGGTAGTGACAATGGCGAGTTTGAGAGGGTATTTATATACTTTGATGAGAATGGAGATGGAAAAGTGTCACCGGCTGAGCTTAGGAAGTGTGTCAAGGCGGTAGGAGGCGAACTGACGGTGGAGGAGGCGGAGATGGCGGTGAGGCTATCGGATTCCGATGGGGATGGATTGTTGGGATTGGAGGATTTTACGAAGTTAATGGAAGGAATGGAAGAGGAGAGGAATAAGGAGAGTGAGTTGATAGGAGCATTTGGAATGTATGAAATGGAGGGGAGTGGCTACATTACTCCTAAGAGATTGAAGAGGATGTTGAGTCAACTCGGTGAGTCAACTTCCATTGACAACTGCAAAACTATGATACGGAGGTTTGATCTAAACGGAGATGGAGTCCTCAGCTTCGATGAATTCAGAGTTATGATGACAACTTAGaagagttcagaaagaaatatttgtgtacataatatgattcttgagctaaaataatcctgagtaacgtttttcagtgagatttttcattttgtagattcatgattgcaaagaactcttggtttcacaatttcacaatttcacaatttaacttggtgctaattctactttcaactttcaacttttgctggaaacggatgctaacttctgcctccgtaaactagaaaaaaatgtctcccttaagctggcgttctggtcaggctgtcacaaggaaagttgcccggatataattggtgttgactatagggagggggggagggggtatcaacctcaaaatacacaaggaattgtccaataaggaaaaactaatttaatggaaaaacaaaagggagtcaaacttcaaactcgaattcaattaggcatgatcaggtttcagagaacagcactctaaatttcagatctagaaaagatcatatgcgtgaattgataggctagaactaggaaaagaggagaggtagactgatgaggctggaaattcagctactcaatgatgctaagttttgctaaggaattaaggtttgtacaaactagctacagcagatcgaggtaatcataatgaaagaaaagtctcagccagaaaaaaaaatggaagtcaaaatccagagatatactttggaaacactaaaggaaagagagattactttctttcagattgattgaacttttcacaagttattcacaatcattaataatagtttgtaatacataatggtttgctagaggagaaattacattgacaatacatgagggcagcatgcaaagagaagatgctagttgtcaattccaagcaacggattcagctcaagcactgaccttcgtatatcatgtagatcctcctctaccttgtcggaaaaagagcatgattggcatgaaaatgcattcagcaactgactttttctggt

> *NbrgsCaM* *6*

Same as the published one

> *NbrgsCaM* *7*

Same as the published one

**Supplementary Data S3. Predicted amino acid sequences of *NbrgsCaMs*.**

> NbrgsCaM 1

M S M K S V S V P S H E S K S V F S R L R N R F S L K K A T P Q K E D K G L T T T T T T A T T D H L S V S S S G S E N S E L D R V F T Y F D E N G D G K V S P T E L R R C V K A V G G E L T M E E V E M A V R L S D S D G D G L L G L E D F T K L M E G M E E E R N K E S E L I G A F G M Y E M E G S G Y I T P K S L K R M L S R L S E S T T I D N C K A M I Q R F D L N G D G V L S F D E F K V M M T S *

> NbrgsCaM 2

M C T I M E S V S V P S A E N K S V F L R L R K R L S L K R Q L L R K K M R V *

M A V R L S D S D G D G L L G L E D F T K L M E G M E E E R N K E S E L I G A F G M Y E M E G S G Y I T P K S L K R M L S Q L G E S T S I D N C K T M I R R F D L N G D G V L S F D E F R V M M T S *

> NbrgsCaM 3

M E T I S S S T E N K S V F S R L M N I F S P K K P I I I K D D E V I D Q T A S T S T L S V S I I N T S I E N S D H L E R V F T Y F D E D G D G K V S P V E L Q R C V R A V G G E L T E E E A E M A V R L S D S D G D G M L G L E D F S K L M E G S D V E E K K K E S E L R G A F E M Y E M E G T G Q I T P K S L K R M L S R L G E S T S I D N C K A M I Q R F D L D G D G V L I F D E F K I M M N M E L K K I *

> NbrgsCaM 4

M E S V S V P S V E N K S Y F S R L N K R F S L K K A T T T T T I T A D R L S M S C S S S R S N N S S E L E R V F T Y F D D N G D G K V S P A E L R R C V K A V G G E L T V E E A E M A V R L S D S D G D G L L G L E D F T K L M E G M E E E R N K E S E L I G A F G M Y E M E G S G Y V T P K S L K R M L S R L G E S T S I D N C K A M I Q R F D I N G D G V L S F D E F K V M M T S *

> NbrgsCaM 5

M C T M E S V S V P S A E N K S V F S R L R K M L S F K K A T P Q K K H E G L T T T N T T T G R L S V S S S G S D N G E F E R V F I Y F D E N G D G K V S P A E L R K C V K A V G G E L T V E E A E M A V R L S D S D G D G L L G L E D F T K L M E G M E E E R N K E S E L I G A F G M Y E M E G S G Y I T P K R L K R M L S Q L G E S T S I D N C K T M I R R F D L N G D G V L S F D E F R V M M T T *

> NbrgsCaM 6

M S M Q S V S V P S H E N K S A F S R L R N R F S L R R K T N V *

M A V R L S D S D G D G L L G L E D F T K L M E G M E E E R N K E S E L I G A F G M Y E M E G S G Y I T P K S L K R M L S Q L G E S T S I D N C K A M I R R F D L N G D G V L S F D E F K V M M T S *

> NbrgsCaM 7

M C M D T T S S S A E N N K S V F S R L R N R F S P K K P I V I K N N D E V I D Q T A S T S T L S V S S E N I D H L E R V F T Y F D E D G D G K V S P A E L Q R C V R A V G G E L T E E E A E M A V R L S D S D G D G M L G L E D I S K L M E G G D V E E K N K G T G L R G A F E M Y E M E R T G Q I T P K S L K R M L N R L G E S T S V D N C K S M I Q R F D L D G D G A L N F D E F K I M M N M E L K N I *

**Supplementary Data S4. Predicted promoter and enhancer elements for *AtrgsCaMs*.**

**> AtrgsCaM37 putative promoter (1208 bp upstream of start codon)**

**2 promoter/enhancer(s) are predicted**

**Promoter Pos: 1158 (TATA box at 1121)**

**Enhancer Pos: 991**

**Transcription factor binding sites/RegSite DB:**

**For promoter at position 1158**

916 (-) RSP00004 tagaCACGTaga

907 (+) RSP00010 cACGTG

904 (+) RSP00011 ctccACGTGgt

1043 (-) RSP00024 TATTATTT

879 (+) RSP00026 gcttttgaTGACtTcaaacac

957 (+) RSP00026 gcttttgaTGACtTcaaacac

908 (+) RSP00065 ACGTGgcgc

908 (+) RSP00066 ACGTGccgc

907 (+) RSP00069 tACGTG

1062 (-) RSP00096 GGTTT

957 (-) RSP00129 CACGAC

1078 (-) RSP00148 CGACG

883 (-) RSP00148 CGACG

1148 (+) RSP00161 WAAAG

1036 (-) RSP00161 WAAAG

867 (-) RSP00161 WAAAG

1067 (-) RSP00162 ACACccGagctaaccacaac

906 (+) RSP00186 cTACGTGgcca

1010 (+) RSP00387 acCGcCGCGCctgc

1060 (-) RSP00398 TTTGAA

1024 (-) RSP00400 CGGTTG

907 (+) RSP00069 TACGTG

912 (-) RSP00427 CACGTA

1080 (-) RSP00431 rdygRCRGTTRs

1029 (-) RSP00431 rdygRCRGTTRs

956 (+) RSP00467 tGGTCAatTc

970 (-) RSP00467 tGGTCAatTc

955 (+) RSP00468 ttGGTCAatTc

971 (-) RSP00468 ttGGTCAatTc

1014 (-) RSP00469 GNGGTG

949 (-) RSP00469 GNGGTG

1099 (-) RSP00470 GTGGNG

978 (-) RSP00470 GTGGNG

1033 (+) RSP00477 TTTAA

1068 (+) RSP00477 TTTAA

1038 (-) RSP00477 TTTAA

1013 (+) RSP00483 GCCGC

922 (-) RSP00508 gcaTTTTTatca

882 (+) RSP00512 cttgtaacCATCAgccaatcgaccagccaatcattc

917 (+) RSP00512 cttgtaacCATCAgccaatcgaccagccaatcattc

1151 (+) RSP00512 cttgtaacCATCAgccaatcgaccagccaatcattc

1072 (-) RSP00518 tccctACACgcGtcacaattc

1076 (-) RSP00519 caattcaggACACgtGccctcttca

1067 (-) RSP00521 ACACccG

1067 (-) RSP00523 ACACgcG

1067 (-) RSP00524 ACACgtG

1083 (+) RSP00613 ATCAAGAA

1075 (-) RSP00630 aagccaagccgccaagTTGatccgtTTGatcgc

**For promoter at position 991**

808 (+) RSP00004 tagaCACGTaga

916 (-) RSP00004 tagaCACGTaga

821 (-) RSP00004 tagaCACGTaga

812 (+) RSP00010 cACGTG

907 (+) RSP00010 cACGTG

817 (-) RSP00010 cACGTG

809 (+) RSP00011 ctccACGTGgt

904 (+) RSP00011 ctccACGTGgt

820 (-) RSP00011 ctccACGTGgt

879 (+) RSP00026 gcttttgaTGACtTcaaacac

957 (+) RSP00026 gcttttgaTGACtTcaaacac

854 (-) RSP00026 gcttttgaTGACtTcaaacac

745 (-) RSP00026 gcttttgaTGACtTcaaacac

813 (+) RSP00065 ACGTGgcgc

908 (+) RSP00065 ACGTGgcgc

816 (-) RSP00065 ACGTGgcgc

813 (+) RSP00066 ACGTGccgc

908 (+) RSP00066 ACGTGccgc

816 (-) RSP00066 ACGTGccgc

812 (+) RSP00069 tACGTG

907 (+) RSP00069 tACGTG

817 (-) RSP00069 tACGTG

854 (+) RSP00096 GGTTT

812 (-) RSP00096 GGTTT

694 (+) RSP00098 cttaatatATTTTTAattattttattctcttaa

698 (+) RSP00117 aattATTTTTAaa

699 (+) RSP00125 aatATTTTTAtt

957 (-) RSP00129 CACGAC

812 (+) RSP00010 CACGTG

817 (-) RSP00010 CACGTG

699 (+) RSP00135 aatATTTTTAtc

698 (+) RSP00140 aattATTTTTAtt

883 (-) RSP00148 CGACG

799 (+) RSP00161 WAAAG

867 (-) RSP00161 WAAAG

818 (-) RSP00162 ACACccGagctaaccacaac

906 (+) RSP00186 cTACGTGgcca

766 (-) RSP00284 CCGTCC

702 (+) RSP00339 RTTTTTR

848 (-) RSP00378 TTTGACT

907 (+) RSP00069 TACGTG

912 (-) RSP00427 CACGTA

759 (+) RSP00431 rdygRCRGTTRs

847 (-) RSP00436 TTGACT

956 (+) RSP00467 tGGTCAatTc

970 (-) RSP00467 tGGTCAatTc

955 (+) RSP00468 ttGGTCAatTc

971 (-) RSP00468 ttGGTCAatTc

949 (-) RSP00469 GNGGTG

748 (+) RSP00470 GTGGNG

978 (-) RSP00470 GTGGNG

705 (+) RSP00477 TTTAA

788 (+) RSP00477 TTTAA

796 (+) RSP00477 TTTAA

801 (-) RSP00477 TTTAA

808 (+) RSP00479 aggaCACGTGtcgc

821 (-) RSP00479 aggaCACGTGtcgc

700 (+) RSP00508 gcaTTTTTatca

922 (-) RSP00508 gcaTTTTTatca

882 (+) RSP00512 cttgtaacCATCAgccaatcgaccagccaatcattc

917 (+) RSP00512 cttgtaacCATCAgccaatcgaccagccaatcattc

823 (-) RSP00518 tccctACACgcGtcacaattc

827 (-) RSP00519 caattcaggACACgtGccctcttca

818 (-) RSP00521 ACACccG

818 (-) RSP00523 ACACgcG

818 (-) RSP00524 ACACgtG

**> AtrgsCaM38 putative promoter (947 bp upstream of start codon)**

**2 promoter/enhancer(s) are predicted**

**Promoter Pos: 875 (TATA box at 839)**

**Promoter Pos: 339 (TATA box at 316)**

**Transcription factor binding sites/RegSite DB:**

**For promoter at position 875**

744 (+) RSP00004 tagaCACGTaga

753 (-) RSP00010 cACGTG

756 (-) RSP00011 ctccACGTGgt

620 (-) RSP00016 caTGCAC

752 (-) RSP00065 ACGTGgcgc

752 (-) RSP00066 ACGTGccgc

753 (-) RSP00069 tACGTG

616 (-) RSP00098 cttaatatATTTTTAattattttattctcttaa

666 (-) RSP00102 aaaTGACGaaaatgc

612 (-) RSP00117 aattATTTTTAaa

611 (-) RSP00125 aatATTTTTAtt

620 (-) RSP00126 gaaaattttaatATTTTTAtttagtatt

611 (-) RSP00135 aatATTTTTAtc

612 (-) RSP00140 aattATTTTTAtt

672 (+) RSP00161 WAAAG

682 (+) RSP00161 WAAAG

717 (+) RSP00161 WAAAG

857 (+) RSP00161 WAAAG

772 (+) RSP00162 ACACccGagctaaccacaac

615 (-) RSP00162 ACACccGagctaaccacaac

678 (-) RSP00269 atcttatgtcattgaTGACGacctcc

677 (-) RSP00305 CCTTTT

608 (-) RSP00339 RTTTTTR

627 (-) RSP00398 TTTGAA

615 (-) RSP00410 ttACGtcAtcgcTtACGcat

753 (-) RSP00423 GACGTG

748 (+) RSP00424 CACGTC

853 (+) RSP00446 ctgtaAAAGtaCTTTtacag

872 (-) RSP00446 ctgtaAAAGtaCTTTtacag

836 (-) RSP00467 tGGTCAatTc

837 (-) RSP00468 ttGGTCAatTc

714 (-) RSP00477 TTTAA

673 (-) RSP00477 TTTAA

605 (-) RSP00477 TTTAA

688 (-) RSP00508 gcaTTTTTatca

678 (-) RSP00508 gcaTTTTTatca

610 (-) RSP00508 gcaTTTTTatca

767 (+) RSP00518 tccctACACgcGtcacaattc

620 (-) RSP00518 tccctACACgcGtcacaattc

763 (+) RSP00519 caattcaggACACgtGccctcttca

624 (-) RSP00519 caattcaggACACgtGccctcttca

772 (+) RSP00521 ACACccG

615 (-) RSP00521 ACACccG

772 (+) RSP00523 ACACgcG

615 (-) RSP00523 ACACgcG

772 (+) RSP00524 ACACgtG

615 (-) RSP00524 ACACgtG

811 (-) RSP00565 GTATTTT

598 (+) RSP00618 CCGTTA

603 (+) RSP00629 AAAAATCT

**For promoter at position 339**

196 (-) RSP00005 CTWWWWWWGT

198 (+) RSP00076 AACGTT

203 (-) RSP00076 AACGTT

67 (+) RSP00161 WAAAG

278 (+) RSP00161 WAAAG

297 (+) RSP00161 WAAAG

146 (-) RSP00161 WAAAG

298 (-) RSP00212 TAATRATTA

54 (-) RSP00241 CGGTCA

328 (-) RSP00339 RTTTTTR

53 (-) RSP00383 GGTCAAA

208 (-) RSP00398 TTTGAA

248 (-) RSP00421 ACCGAGA

266 (-) RSP00431 rdygRCRGTTRs

245 (-) RSP00444 aagAAAGAAAGaaa

112 (+) RSP00477 TTTAA

300 (-) RSP00477 TTTAA

70 (-) RSP00477 TTTAA

40 (+) RSP00508 gcaTTTTTatca

41 (+) RSP00508 gcaTTTTTatca

42 (+) RSP00508 gcaTTTTTatca

140 (+) RSP00508 gcaTTTTTatca

147 (+) RSP00508 gcaTTTTTatca

148 (+) RSP00508 gcaTTTTTatca

149 (+) RSP00508 gcaTTTTTatca

150 (+) RSP00508 gcaTTTTTatca

151 (+) RSP00508 gcaTTTTTatca

152 (+) RSP00508 gcaTTTTTatca

330 (-) RSP00508 gcaTTTTTatca

284 (-) RSP00508 gcaTTTTTatca

283 (-) RSP00508 gcaTTTTTatca

282 (-) RSP00508 gcaTTTTTatca

281 (-) RSP00508 gcaTTTTTatca

280 (-) RSP00508 gcaTTTTTatca

279 (-) RSP00508 gcaTTTTTatca

278 (-) RSP00508 gcaTTTTTatca

277 (-) RSP00508 gcaTTTTTatca

276 (-) RSP00508 gcaTTTTTatca

275 (-) RSP00508 gcaTTTTTatca

274 (-) RSP00508 gcaTTTTTatca

182 (-) RSP00508 gcaTTTTTatca

52 (+) RSP00618 CCGTTA

**> AtrgsCaM39 putative promoter (849 bp upstream of start codon)**

**1 promoter/enhancer(s) are predicted**

**Promoter Pos: 773 (TATA box at 738)**

**Transcription factor binding sites/RegSite DB:**

**For promoter at position 773**

679 (+) RSP00004 tagaCACGTaga

544 (-) RSP00004 tagaCACGTaga

535 (+) RSP00010 cACGTG

688 (-) RSP00010 cACGTG

532 (+) RSP00011 ctccACGTGgt

691 (-) RSP00011 ctccACGTGgt

536 (+) RSP00065 ACGTGgcgc

687 (-) RSP00065 ACGTGgcgc

536 (+) RSP00066 ACGTGccgc

687 (-) RSP00066 ACGTGccgc

535 (+) RSP00069 tACGTG

688 (-) RSP00069 tACGTG

659 (-) RSP00098 cttaatatATTTTTAattattttattctcttaa

655 (-) RSP00117 aattATTTTTAaa

654 (-) RSP00125 aatATTTTTAtt

663 (-) RSP00126 gaaaattttaatATTTTTAtttagtatt

654 (-) RSP00135 aatATTTTTAtc

655 (-) RSP00140 aattATTTTTAtt

638 (+) RSP00161 WAAAG

764 (-) RSP00161 WAAAG

759 (+) RSP00265 ACTTTA

477 (-) RSP00269 atcttatgtcattgaTGACGacctcc

570 (+) RSP00316 AACCAA

651 (-) RSP00339 RTTTTTR

647 (-) RSP00402 TTAGTCT

616 (-) RSP00402 TTAGTCT

509 (+) RSP00477 TTTAA

542 (+) RSP00477 TTTAA

547 (-) RSP00477 TTTAA

595 (+) RSP00503 CTAGCTAG

602 (-) RSP00503 CTAGCTAG

654 (+) RSP00508 gcaTTTTTatca

655 (+) RSP00508 gcaTTTTTatca

656 (+) RSP00508 gcaTTTTTatca

657 (+) RSP00508 gcaTTTTTatca

658 (+) RSP00508 gcaTTTTTatca

659 (+) RSP00508 gcaTTTTTatca

653 (-) RSP00508 gcaTTTTTatca

**Supplementary Data S5. Predicted promoter and enhancer elements for *NbrgsCaMs*.**

**> NbrgsCaM1 putative promoter (1 kbp upstream of start codon)**

**2 promoter/enhancer(s) are predicted**

**Promoter Pos: 856 (TATA box at 826)**

**Promoter Pos: 139 (TATA box at 105)**

**Transcription factor binding sites/RegSite DB:**

**For promoter at position 856**

704 (+) RSP00005 CTWWWWWWGT

596 (-) RSP00026 gcttttgaTGACtTcaaacac

581 (+) RSP00161 WAAAG

630 (+) RSP00161 WAAAG

766 (-) RSP00161 WAAAG

688 (-) RSP00161 WAAAG

682 (-) RSP00161 WAAAG

676 (-) RSP00162 ACACccGagctaaccacaac

613 (+) RSP00227 AAGATAAGA

838 (-) RSP00337 ATATTTAWW

706 (+) RSP00339 RTTTTTR

777 (-) RSP00397 AGTGGCGG

764 (+) RSP00398 TTTGAA

692 (-) RSP00398 TTTGAA

560 (-) RSP00398 TTTGAA

757 (+) RSP00422 ACCGAC

589 (-) RSP00436 TTGACT

749 (+) RSP00463 atttcatggCCGACctgcttttt

774 (+) RSP00463 atttcatggCCGACctgcttttt

749 (+) RSP00464 acttgatggCCGACctctttttt

774 (+) RSP00464 acttgatggCCGACctctttttt

749 (+) RSP00465 aatatactaCCGACcatgagttct

774 (+) RSP00465 aatatactaCCGACcatgagttct

754 (+) RSP00466 actaCCGACatgagttccaaaaagc

779 (+) RSP00466 actaCCGACatgagttccaaaaagc

776 (-) RSP00470 GTGGNG

578 (+) RSP00477 TTTAA

716 (+) RSP00477 TTTAA

583 (-) RSP00477 TTTAA

734 (+) RSP00502 TACGTA

739 (-) RSP00502 TACGTA

704 (+) RSP00508 gcaTTTTTatca

641 (+) RSP00512 cttgtaacCATCAgccaatcgaccagccaatcattc

815 (+) RSP00512 cttgtaacCATCAgccaatcgaccagccaatcattc

681 (-) RSP00518 tccctACACgcGtcacaattc

685 (-) RSP00519 caattcaggACACgtGccctcttca

676 (-) RSP00521 ACACccG

676 (-) RSP00523 ACACgcG

676 (-) RSP00524 ACACgtG

712 (+) RSP00565 GTATTTT

758 (-) RSP00565 GTATTTT

573 (+) RSP00625 aaggtgatcatagagcaTATTATAagagagtgaaaactaatgg

584 (-) RSP00630 aagccaagccgccaagTTGatccgtTTGatcgc

**For promoter at position 139**

87 (+) RSP00398 TTTGAA

10 (+) RSP00402 TTAGTCT

59 (+) RSP00477 TTTAA

**> NbrgsCaM3 putative promoter (1 kbp upstream of start codon)**

**1 promoter/enhancer(s) are predicted**

**Promoter Pos: 858 (TATA box at 825)**

**Transcription factor binding sites/RegSite DB:**

**For promoter at position 858**

566 (+) RSP00004 tagaCACGTaga

575 (-) RSP00010 cACGTG

578 (-) RSP00011 ctccACGTGgt

644 (+) RSP00026 gcttttgaTGACtTcaaacac

574 (-) RSP00065 ACGTGgcgc

574 (-) RSP00066 ACGTGccgc

575 (-) RSP00069 tACGTG

715 (-) RSP00096 GGTTT

638 (-) RSP00127 AGCGGG

670 (+) RSP00161 WAAAG

677 (+) RSP00161 WAAAG

780 (-) RSP00161 WAAAG

762 (-) RSP00161 WAAAG

659 (-) RSP00161 WAAAG

652 (-) RSP00161 WAAAG

647 (-) RSP00161 WAAAG

682 (-) RSP00254 CCTTTTC

757 (+) RSP00265 ACTTTA

775 (+) RSP00265 ACTTTA

781 (+) RSP00304 TTTTTTCC

682 (-) RSP00305 CCTTTT

683 (+) RSP00308 CAACA

605 (+) RSP00337 ATATTTAWW

712 (+) RSP00338 AACCCA

836 (+) RSP00338 AACCCA

650 (+) RSP00378 TTTGACT

576 (-) RSP00401 TAACGT

771 (-) RSP00431 rdygRCRGTTRs

651 (+) RSP00436 TTGACT

745 (+) RSP00463 atttcatggCCGACctgcttttt

745 (+) RSP00464 acttgatggCCGACctctttttt

745 (+) RSP00465 aatatactaCCGACcatgagttct

750 (+) RSP00466 actaCCGACatgagttccaaaaagc

634 (-) RSP00469 GNGGTG

657 (+) RSP00477 TTTAA

759 (+) RSP00477 TTTAA

814 (-) RSP00477 TTTAA

578 (-) RSP00477 TTTAA

778 (+) RSP00508 gcaTTTTTatca

779 (+) RSP00508 gcaTTTTTatca

676 (-) RSP00508 gcaTTTTTatca

767 (+) RSP00618 CCGTTA

579 (+) RSP00625 aaggtgatcatagagcaTATTATAagagagtgaaaactaatgg

**> NbrgsCaM4 putative promoter (1 kbp upstream of start codon)**

**1 promoter/enhancer(s) are predicted**

**Promoter Pos: 873 (TATA box at 839)**

**Transcription factor binding sites/RegSite DB:**

**For promoter at position 873**

786 (-) RSP00004 tagaCACGTaga

777 (+) RSP00010 cACGTG

774 (+) RSP00011 ctccACGTGgt

778 (+) RSP00065 ACGTGgcgc

778 (+) RSP00066 ACGTGccgc

777 (+) RSP00069 tACGTG

605 (+) RSP00093 TATCCAC

678 (-) RSP00096 GGTTT

667 (-) RSP00096 GGTTT

584 (-) RSP00102 aaaTGACGaaaatgc

806 (+) RSP00122 CTCCCAC

715 (-) RSP00148 CGACG

858 (-) RSP00161 WAAAG

742 (-) RSP00161 WAAAG

689 (-) RSP00161 WAAAG

776 (+) RSP00186 cTACGTGgcca

593 (+) RSP00227 AAGATAAGA

853 (+) RSP00254 CCTTTTC

618 (+) RSP00100 TGTCTC

596 (-) RSP00269 atcttatgtcattgaTGACGacctcc

684 (+) RSP00305 CCTTTT

853 (+) RSP00305 CCTTTT

648 (-) RSP00308 CAACA

851 (-) RSP00337 ATATTTAWW

677 (-) RSP00339 RTTTTTR

665 (-) RSP00398 TTTGAA

708 (+) RSP00401 TAACGT

777 (+) RSP00069 TACGTG

782 (-) RSP00427 CACGTA

826 (-) RSP00469 GNGGTG

653 (+) RSP00477 TTTAA

694 (+) RSP00477 TTTAA

683 (+) RSP00508 gcaTTTTTatca

684 (+) RSP00508 gcaTTTTTatca

685 (+) RSP00508 gcaTTTTTatca

686 (+) RSP00508 gcaTTTTTatca

687 (+) RSP00508 gcaTTTTTatca

688 (+) RSP00508 gcaTTTTTatca

689 (+) RSP00508 gcaTTTTTatca

736 (+) RSP00508 gcaTTTTTatca

679 (-) RSP00508 gcaTTTTTatca

617 (+) RSP00512 cttgtaacCATCAgccaatcgaccagccaatcattc

626 (+) RSP00512 cttgtaacCATCAgccaatcgaccagccaatcattc

861 (+) RSP00512 cttgtaacCATCAgccaatcgaccagccaatcattc

598 (-) RSP00630 aagccaagccgccaagTTGatccgtTTGatcgc

**> NbrgsCaM5 putative promoter (1 kbp upstream of start codon)**

**2 promoter/enhancer(s) are predicted**

**Promoter Pos: 859 (TATA box at 824)**

**Promoter Pos: 375 (TATA box at 341)**

**Transcription factor binding sites/RegSite DB:**

**For promoter at position 859**

690 (+) RSP00005 CTWWWWWWGT

754 (-) RSP00161 WAAAG

667 (-) RSP00161 WAAAG

569 (+) RSP00162 ACACccGagctaaccacaac

599 (+) RSP00227 AAGATAAGA

662 (+) RSP00305 CCTTTT

589 (-) RSP00308 CAACA

586 (-) RSP00308 CAACA

836 (-) RSP00337 ATATTTAWW

692 (+) RSP00339 RTTTTTR

678 (-) RSP00398 TTTGAA

745 (+) RSP00422 ACCGAC

737 (+) RSP00463 atttcatggCCGACctgcttttt

737 (+) RSP00464 acttgatggCCGACctctttttt

737 (+) RSP00465 aatatactaCCGACcatgagttct

742 (+) RSP00466 actaCCGACatgagttccaaaaagc

661 (+) RSP00508 gcaTTTTTatca

690 (+) RSP00508 gcaTTTTTatca

698 (+) RSP00508 gcaTTTTTatca

627 (+) RSP00512 cttgtaacCATCAgccaatcgaccagccaatcattc

564 (+) RSP00518 tccctACACgcGtcacaattc

560 (+) RSP00519 caattcaggACACgtGccctcttca

569 (+) RSP00521 ACACccG

569 (+) RSP00523 ACACgcG

569 (+) RSP00524 ACACgtG

698 (+) RSP00565 GTATTTT

746 (-) RSP00565 GTATTTT

**For promoter at position 375**

129 (-) RSP00016 caTGCAC

347 (-) RSP00022 ATAATAAAA

186 (-) RSP00026 gcttttgaTGACtTcaaacac

246 (+) RSP00092 TAACAAA

283 (+) RSP00096 GGTTT

312 (-) RSP00096 GGTTT

353 (+) RSP00161 WAAAG

120 (-) RSP00304 TTTTTTCC

358 (-) RSP00305 CCTTTT

334 (+) RSP00308 CAACA

276 (+) RSP00339 RTTTTTR

305 (-) RSP00339 RTTTTTR

187 (-) RSP00398 TTTGAA

205 (-) RSP00469 GNGGTG

327 (-) RSP00470 GTGGNG

285 (+) RSP00477 TTTAA

302 (-) RSP00477 TTTAA

153 (-) RSP00477 TTTAA

274 (+) RSP00508 gcaTTTTTatca

359 (-) RSP00508 gcaTTTTTatca

358 (-) RSP00508 gcaTTTTTatca

357 (-) RSP00508 gcaTTTTTatca

356 (-) RSP00508 gcaTTTTTatca

355 (-) RSP00508 gcaTTTTTatca

307 (-) RSP00508 gcaTTTTTatca

124 (-) RSP00508 gcaTTTTTatca

123 (-) RSP00508 gcaTTTTTatca

122 (-) RSP00508 gcaTTTTTatca

121 (-) RSP00512 cttgtaacCATCAgccaatcgaccagccaatcattc

198 (+) RSP00615 CTCACC

325 (+) RSP00625 aaggtgatcatagagcaTATTATAagagagtgaaaactaatgg

**> NbrgsCaM7 putative promoter (1 kbp upstream of start codon)**

**2 promoter/enhancer(s) are predicted**

**Promoter Pos: 827 (TATA box at 790)**

**Enhancer Pos: 599**

**Transcription factor binding sites/RegSite DB:**

**For promoter at position 827**

611 (+) RSP00003 CCWWWWWWRG

576 (+) RSP00026 gcttttgaTGACtTcaaacac

589 (+) RSP00026 gcttttgaTGACtTcaaacac

658 (-) RSP00096 GGTTT

581 (+) RSP00102 aaaTGACGaaaatgc

616 (+) RSP00161 WAAAG

621 (+) RSP00161 WAAAG

745 (-) RSP00161 WAAAG

604 (-) RSP00161 WAAAG

597 (-) RSP00161 WAAAG

541 (-) RSP00161 WAAAG

579 (+) RSP00162 ACACccGagctaaccacaac

584 (+) RSP00214 TGACGTgg

626 (-) RSP00254 CCTTTTC

569 (+) RSP00269 atcttatgtcattgaTGACGacctcc

746 (+) RSP00304 TTTTTTCC

626 (-) RSP00305 CCTTTT

627 (+) RSP00308 CAACA

570 (-) RSP00337 ATATTTAWW

655 (+) RSP00338 AACCCA

801 (+) RSP00338 AACCCA

595 (+) RSP00378 TTTGACT

709 (-) RSP00400 CGGTTG

714 (-) RSP00431 rdygRCRGTTRs

596 (+) RSP00436 TTGACT

589 (-) RSP00437 ACGTCA

688 (+) RSP00463 atttcatggCCGACctgcttttt

688 (+) RSP00464 acttgatggCCGACctctttttt

688 (+) RSP00465 aatatactaCCGACcatgagttct

693 (+) RSP00466 actaCCGACatgagttccaaaaagc

602 (+) RSP00477 TTTAA

779 (-) RSP00477 TTTAA

567 (-) RSP00477 TTTAA

582 (-) RSP00486 atactaattattaTATTTAAT

582 (-) RSP00487 atattaattagaaTATTTAAT

743 (+) RSP00508 gcaTTTTTatca

744 (+) RSP00508 gcaTTTTTatca

622 (-) RSP00508 gcaTTTTTatca

621 (-) RSP00508 gcaTTTTTatca

574 (+) RSP00518 tccctACACgcGtcacaattc

570 (+) RSP00519 caattcaggACACgtGccctcttca

579 (+) RSP00521 ACACccG

579 (+) RSP00523 ACACgcG

579 (+) RSP00524 ACACgtG

**For promoter at position 599**

469 (-) RSP00024 TATTATTT

381 (+) RSP00026 gcttttgaTGACtTcaaacac

576 (+) RSP00026 gcttttgaTGACtTcaaacac

589 (+) RSP00026 gcttttgaTGACtTcaaacac

435 (-) RSP00026 gcttttgaTGACtTcaaacac

421 (-) RSP00029 TGTAAAAG

321 (+) RSP00092 TAACAAA

581 (+) RSP00102 aaaTGACGaaaatgc

514 (+) RSP00161 WAAAG

597 (-) RSP00161 WAAAG

541 (-) RSP00161 WAAAG

418 (-) RSP00161 WAAAG

342 (-) RSP00161 WAAAG

579 (+) RSP00162 ACACccGagctaaccacaac

362 (+) RSP00212 TAATRATTA

373 (-) RSP00212 TAATRATTA

584 (+) RSP00214 TGACGTgg

569 (+) RSP00269 atcttatgtcattgaTGACGacctcc

570 (-) RSP00337 ATATTTAWW

595 (+) RSP00378 TTTGACT

596 (+) RSP00436 TTGACT

589 (-) RSP00437 ACGTCA

394 (-) RSP00467 tGGTCAatTc

395 (-) RSP00468 ttGGTCAatTc

567 (-) RSP00477 TTTAA

582 (-) RSP00486 atactaattattaTATTTAAT

582 (-) RSP00487 atattaattagaaTATTTAAT

336 (+) RSP00508 gcaTTTTTatca

526 (-) RSP00508 gcaTTTTTatca

574 (+) RSP00518 tccctACACgcGtcacaattc

570 (+) RSP00519 caattcaggACACgtGccctcttca

579 (+) RSP00521 ACACccG

579 (+) RSP00523 ACACgcG

579 (+) RSP00524 ACACgtG

353 (+) RSP00593 GAATCTTAT

352 (+) RSP00625 aaggtgatcatagagcaTATTATAagagagtgaaaactaatgg

525 (+) RSP00625 aaggtgatcatagagcaTATTATAagagagtgaaaactaatgg

**Supplementary Figure S1. *NbrgsCaM2* and *NbrgsCaM6* are putative pseudogenes.**

Schematic view of the amino acid sequences deduced from *NbrgsCaM2* and *NbrgsCaM6.* The deduced short and long amino acid sequences of NbrgsCaM2/6 align only partly with NbrgsCaM4, which has two EF-hand motifs.





**Supplementary Figure S2. Vascular and phloem tissues occupy most of the leaf in *βC1* transgenic *N. benthamiana*.**

Sections of leaves from the WT and *βC1* transgenic *N. benthamiana* plants. The *βC1* transgenic leaves are deformed with vascular and phloem tissues occupy a far larger portion of the section than WT.


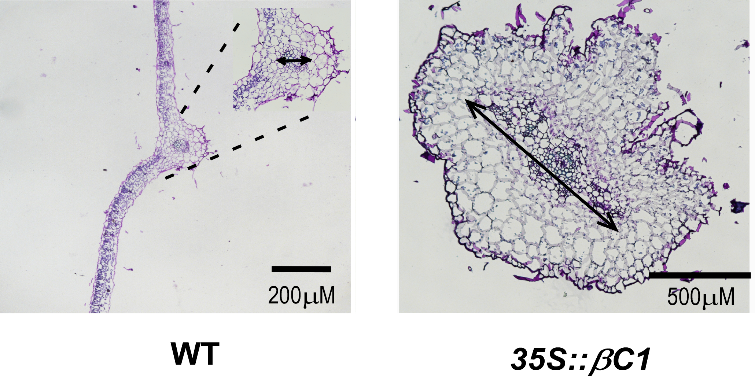


**Supplementary Table S1. Transcription factor for the predicted enhancer motifs in *AtrgsCaMp*s.**

| *AtCMLp*  Predicted enhancer  binding transcription factors | 37 | 38 | 39 |
| --- | --- | --- | --- |
| Alfin1 | √ |  |  |
| AT1 | √ | √ | √ |
| ATHB-2 |  | √ |  |
| AtMyb77 | √ | √ |  |
| CCA1 | √ | √ |  |
| Dof1 | √ | √ | √ |
| DPBF-1,-2 | √ | √ |  |
| EmBP-1 | √ | √ | √ |
| Epicotyl-specific nuclear factor | √ | √ | √ |
| NtBBF1 |  |  | √ |
| Obf5 |  | √ | √ |
| Root-specific nuclear factor | √ | √ | √ |
| SEF4 | √ | √ | √ |
| TAF-1 | √ | √ | √ |
| WRKY1 | √ | √ |  |

**Supplementary Table S2. Transcription factor for the predicted enhancer motifs in *NbrgsCaMp*s.**

| *NbrgsCaMp*  Predicted enhancer  binding transcription factors | 1 | 3 | 4 | 5 | 7 |
| --- | --- | --- | --- | --- | --- |
| AGL3 ＊ | √ |  |  | √ |  |
| Alfin1 ◆ | √ | √ | √ | √ |  |
| AT1 ◎ |  |  |  |  |  |
| ATHB-2 ◆ |  |  |  |  | √ |
| AtMyb77 ◆ | √ | √ |  |  | √ |
| CBF1 ＊ | √ | √ |  | √ | √ |
| CCA1 ◆ | √ |  |  |  |  |
| Dof1 ◆ | √ | √ | √ | √ | √ |
| DPBF-1,-2 ◆ | √ |  |  | √ | √ |
| EmBP-1 ◆ |  | √ | √ |  |  |
| Epicotyl-specific nuclear factor ◆ |  | √ | √ |  |  |
| GBF1 ＊ |  |  |  |  | √ |
| GT-1 ＊ | √ |  | √ | √ |  |
| NtBBF1 ◆ |  | √ |  |  |  |
| Obf5 ◆ |  |  | √ |  | √ |
| RAV1 ＊ |  | √ | √ | √ | √ |
| Root-specific nuclear factor ◆ |  | √ | √ |  |  |
| SEF4 ◎ |  |  |  |  |  |
| TAF-1 ◆ |  | √ | √ |  |  |
| TGA1 ＊ |  | √ |  | √ | √ |
| WRKY1 ◆ |  | √ |  |  | √ |

◎ Predicted enhancer binding transcription factors for *AtrgsCaMp*s (promoters of *AtCML37,38,39*) only;

＊ For *NbrgsCaMp* only;

◆ For both *AtrgsCaMp* and *NbrgsCaMp*

Transcription factors for the enhancer elements in *AtrgsCaMp*s and *NbrgsCaMp*s.

AGL3: Development of flower.

Alfin1: Increases plant growth and salt tolerance [^1^](#_ENREF_1).

AT-1: Response to light [^2^](#_ENREF_2)

ATHB-2: Response to auxin, response to cytokinin, root development, shade avoidance, shoot system morphogenesis, unidimensional cell growth. Meristem development [^3^](#_ENREF_3)

AtMyb77: Cell differentiation, lateral root development, response to chitin, response to ethylene, response to salicylic acid.

CBF1: Cold acclimation, response to cold, response to water deprivation.

CCA1: Circadian rhythm, negative regulation of circadian rhythm, , response to abscisic acid, response to auxin, response to cadmium ion, response to cold, response to ethylene, response to gibberellin, response to jasmonic acid, response to organonitrogen compound, response to salicylic acid, response to salt stress [^4^](#_ENREF_4).

Dof1: Response to chitin.

DPBF-1,-2: Its expression is normally embryo-specific and also can be induced by abscisic acid [^5^](#_ENREF_5).

EmBP-1: Alters vegetative development.

Epicotyl-specific nuclear factor:

GBF1: Regulation of cell aging, regulation of hydrogen peroxide metabolic process.

GT-1:Light-responsive [^6^](#_ENREF_6).

NtBBF1: Auxin-regulated expression of the rolB oncogene in plants [^7^](#_ENREF_7).

Obf5: Defense response, response to xenobiotic stimulus, salicylic acid mediated signaling pathway [^8^](#_ENREF_8).

RAV1: Ethylene-activated signaling pathway, lateral root development, leaf development, negative regulation of flower development, response to brassinosteroid.

Root-specific nuclear factor(s): Phloem-specific functions in vascular development.

SEF4: Seed specific[^9^](#_ENREF_9)

TAF-1: Seed-specific.

TGA1: Defense response to bacterium.

WRKY1: Salicylic acid mediated signaling pathway.

**Supplementary Table S3.** **Developmental, environmental and plant hormonal regulations that *NbrgsCaMp*s are predicted to be involved in.**

| *NbrgsCaMp*  Types of regulation | | 1 | 3 | 4 | 5 | 7 |
| --- | --- | --- | --- | --- | --- | --- |
| Development | Seed-specific |  | √ | √ |  |  |
|  | Epicotyl-specific |  | √ | √ |  |  |
|  | Leaf development |  | √ | √ | √ | √ |
|  | Root-specific | √ | √ | √ | √ | √ |
|  | Vegetative growth | √ | √ | √ | √ |  |
|  | Flower development | √ | √ | √ | √ | √ |
| Environment | Salt stress | √ | √ | √ | √ |  |
|  | Cold | √ | √ |  | √ | √ |
|  | Water deprivation | √ | √ |  | √ | √ |
|  | Light | √ |  | √ | √ | √ |
|  | Hydrogen peroxide metabolic |  |  |  |  | √ |
|  | Chitin | √ | √ | √ | √ | √ |
|  | Defense |  | √ | √ | √ | √ |
| Plant hormones | Abscisic acid | √ |  |  | √ | √ |
|  | Auxin | √ | √ |  |  | √ |
|  | Brassinosteroid |  | √ | √ | √ | √ |
|  | Cytokinin |  |  |  |  | √ |
|  | Ethylene | √ | √ | √ | √ | √ |
|  | Gibberellin | √ |  |  |  |  |
|  | Jasmonic acid | √ |  |  |  |  |
|  | Salicylic acid | √ | √ | √ |  | √ |

**Reference**

1 Winicov, I. Alfin1 transcription factor overexpression enhances plant root growth under normal and saline conditions and improves salt tolerance in alfalfa. *Planta* **210**, 416-422, doi:10.1007/PL00008150 (2000).

2 Datta, N. & Cashmore, A. R. Binding of a pea nuclear protein to promoters of certain photoregulated genes is modulated by phosphorylation. *The Plant cell* **1**, 1069-1077, doi:10.1105/tpc.1.11.1069 (1989).

3 Turchi, L. *et al.* Arabidopsis HD-Zip II transcription factors control apical embryo development and meristem function. *Development* **140**, 2118-2129, doi:10.1242/dev.092833 (2013).

4 Nagel, D. H. *et al.* Genome-wide identification of CCA1 targets uncovers an expanded clock network in Arabidopsis. *Proceedings of the National Academy of Sciences of the United States of America* **112**, E4802-4810, doi:10.1073/pnas.1513609112 (2015).

5 Kim, S. Y., Chung, H. J. & Thomas, T. L. Isolation of a novel class of bZIP transcription factors that interact with ABA-responsive and embryo-specification elements in the Dc3 promoter using a modified yeast one-hybrid system. *The Plant journal : for cell and molecular biology* **11**, 1237-1251 (1997).

6 Green, P. J. *et al.* Binding site requirements for pea nuclear protein factor GT-1 correlate with sequences required for light-dependent transcriptional activation of the rbcS-3A gene. *The EMBO journal* **7**, 4035-4044 (1988).

7 Baumann, K., De Paolis, A., Costantino, P. & Gualberti, G. The DNA binding site of the Dof protein NtBBF1 is essential for tissue-specific and auxin-regulated expression of the rolB oncogene in plants. *The Plant cell* **11**, 323-334 (1999).

8 Foley, R. C. *et al.* Isolation of a maize bZIP protein subfamily: candidates for the ocs-element transcription factor. *The Plant journal : for cell and molecular biology* **3**, 669-679 (1993).

9 Fujiwara, T. & Beachy, R. N. Tissue-specific and temporal regulation of a beta-conglycinin gene: roles of the RY repeat and other cis-acting elements. *Plant molecular biology* **24**, 261-272 (1994).

**Supplementary Table S4. Primers used in this study.**

| Purposes | Primer names | Sequences | Restriction enzymes used in vector construction |
| --- | --- | --- | --- |
| For the construction of pBI101.NbrgsCamp::GUS | p1F | TCTAGA ATATCGATTTGGTCTGCCTC | XbaI |
|  | p1R | CCCGGG GCTAACAAACGCGTTCAGA | SmaI |
|  | p3F | GTCGAC AGTTACGGTCTCGTCACAAC | SalI |
|  | p3R | CCCGGG GCAAATGCTATATGGAAAC | SmaI |
|  | p4F | AAGCTT CTGCACCCAAGGCTATGAT | HindIII |
|  | p4R | GGATCC GCACATATTGCACGTATA | BamHI |
|  | p5F | AAGCTT TCCACCAAACCCAAATTC | HindIII |
|  | p5R | GGATCC GCTAACAACCACGTTTAG | BamHI |
|  | p7F | GTCGAC CTCACAAGGTCCTGGCAA | SalI |
|  | p7R | CCCGGG GCTATATGCAAACTCCGG | SmaI |
| For the cloning of the coding sequences of NbrgsCaMs | Nbrgs1 R | ACAGCTGGAGTCCCCTTGGCATCAT |  |
|  | Nbrgs1 F | TGCTCCCCTTCCTCACTCATCATA |  |
|  | Nbrgs2 R | ACTAGCTATCGCTTTTGCTTTGCATC |  |
|  | Nbrgs2 F | CCGTACAAGTTTCTATACCG |  |
|  | Nbrgs3 R | CTCAATCTCAGCTGTCTTCTTC |  |
|  | Nbrgs3 F | TCTTTGGAAACCCACAAGCCAACT |  |
|  | Nbrgs4 R | GGATAATACAAAGCTGGAGTTCCCTTGGC |  |
|  | Nbrgs4 F | GAAGAATGACCTACCTCTCCCTGCT |  |
|  | Nbrgs5 R | CTCCTCTTTTCCTAGTTCTAGCCTATC |  |
|  | Nbrgs5 F | CTCCAGCTCACAAACTAAATGTTCC |  |
|  | Nbrgs6 R | TTCGTGCATTGCGCGAGTTCAAA |  |
|  | Nbrgs6 F | AAGTTCCGGTCTCTTCGAATTGC |  |
|  | Nbrgs7 R | ATCTGGATTGTTACAAGCGCGGACT |  |
|  | Nbrgs7 F | ACTAACCTCTTCCCCATTACGACTTC |  |
| qPCR primers | qp GAPDH F | ACTACTGTCCACTCCCTTACT |  |
|  | qp GAPDH R | GGTCCACAACAGAAACATCAAC |  |
|  | qp GUS F | GAATACGGCGTGGATACGTTAG |  |
|  | qp GUS R | GATCAAAGACGCGGTGATACA |  |
|  | qp Nbrgs4 F | GGCAACGACAACAACAACTATT |  |
|  | qp Nbrgs4 R | ACCCTCTCTAACTCGCTACTATT |  |
